# The BMP antagonist gremlin 1 contributes to the development of cortical excitatory neurons, motor balance and fear responses

**DOI:** 10.1242/dev.195883

**Published:** 2021-07-12

**Authors:** Mari Ichinose, Nobumi Suzuki, Tongtong Wang, Hiroki Kobayashi, Laura Vrbanac, Jia Q. Ng, Josephine A. Wright, Tamsin R. M. Lannagan, Krystyna A. Gieniec, Martin Lewis, Ryota Ando, Atsushi Enomoto, Simon Koblar, Paul Thomas, Daniel L. Worthley, Susan L. Woods

**Affiliations:** 1 School of Medicine, Faculty of Health and Medical Sciences, University of Adelaide, SA 5000, Australia; 2 Precision Medicine, South Australian Health and Medical Research Institute, Adelaide, SA 5000, Australia; 3 Department of Pathology, Nagoya University Graduate School of Medicine, Nagoya 466-8560, Japan; 4 Department of Psychiatry, College of Medicine and Public Health, Flinders University, Bedford Park, SA 5001, Australia; 5 Lifelong Health, South Australian Health and Medical Research Institute, Adelaide, SA 5000, Australia

**Keywords:** Grem1, BMP, Cortical development, Excitatory neuron, Mouse

## Abstract

Bone morphogenetic protein (BMP) signaling is required for early forebrain development and cortical formation. How the endogenous modulators of BMP signaling regulate the structural and functional maturation of the developing brain remains unclear. Here, we show that expression of the BMP antagonist *Grem1* marks committed layer V and VI glutamatergic neurons in the embryonic mouse brain. Lineage tracing of *Grem1*-expressing cells in the embryonic brain was examined by administration of tamoxifen to pregnant *Grem1creERT*; *Rosa26LSLTdtomato* mice at 13.5 days post coitum (dpc), followed by collection of embryos later in gestation. In addition, at 14.5 dpc, bulk mRNA-seq analysis of differentially expressed transcripts between FACS-sorted *Grem1*-positive and -negative cells was performed. We also generated *Emx1-cre*-mediated *Grem1* conditional knockout mice (*Emx1-Cre;Grem1^flox/flox^*) in which the *Grem1* gene was deleted specifically in the dorsal telencephalon. *Grem1^Emx1cKO^* animals had reduced cortical thickness, especially layers V and VI, and impaired motor balance and fear sensitivity compared with littermate controls. This study has revealed new roles for Grem1 in the structural and functional maturation of the developing cortex.

## Abstract (precis)

**Summary:** The BMP antagonist *Grem1* is expressed by committed deep-layer glutamatergic neurons in the embryonic mouse cortex. *Grem1* conditional knockout mice display cortical and behavioral abnormalities.

## INTRODUCTION

During development of the nervous system, bone morphogenetic protein (BMP) signaling has important roles in the promotion of dorsal identity, and the regulation of cell proliferation and differentiation. Yet BMP function in the maturation of neurons *in vivo* is poorly understood. Misregulation of BMP signaling has been suggested to contribute to human neurodevelopmental conditions such as autism spectrum disorders (Yamasaki et al., 2015; Chen et al., 2017); however, a detailed understanding of the exact role for BMP signaling in these disorders is lacking, in line with our imperfect knowledge of the regulation of the BMP pathway in normal brain development.

The BMP ligands (BMP2/4/5/6/7) bind to type I (BmprIA, BmprIB, Acvr1) and type II (BmprII, ActrIIA, and ActrIIB) receptors. BMP ligands are expressed broadly in the telencephalon during early gestation in mice and then become more tightly localized to the choroid plexus at 13.5 days post coitum (dpc) (Furuta et al., 1997). BMPs have also been derived from the mesenchyme of the brain, such as meninges and endothelial cells (Imura et al., 2008; Choe and Pleasure, 2018). Both type I and II BMP receptors are expressed in the telencephalon during development, but this is restricted in adulthood to BmprII in the cortex and hippocampus and ActrIIA/IIB in the dentate gyrus (Söderström et al., 1996). Aside from this spatiotemporal regulation of receptors, the BMP signaling pathway is also regulated by a family of secreted extracellular antagonists that directly bind to the BMP ligands to prevent interactions with BMP receptors both in development and disease (Ali and Brazil, 2014). Antagonists such as gremlin 1 (Grem1), noggin (Nog) and chordin (Chrd) have been shown to inhibit BMP action in a range of different cell types and developmental stage-specific contexts to provide exquisite spatiotemporal regulation of the pathway. The roles of Nog and Chrd have been partially elucidated: they are required for forebrain development (Bachiller et al., 2000), as well as to create a niche for adult hippocampal neurogenesis (Lim et al., 2000; Sun et al., 2007). The expression and function of Grem1 in the developing brain has not yet been determined. Grem1 is an extracellular secreted antagonist of BMP2/4/7 that signals to intestinal stem cells in the gut (Worthley et al., 2015) and plays a crucial role in *Xenopus* dorsalization (Hsu et al., 1998) and limb and kidney formation (Khokha et al., 2003). Likewise the role of Grem1 in the normal adult CNS is unmapped territory to date, outside of previous work in the pathogenic state of glioma (Yan et al., 2014; Guan et al., 2017; Fu et al., 2018).

BMP signaling has been implicated in the regulation of forebrain patterning during early embryogenesis, working together with other signaling pathways such as fibroblast growth factor (FGF), Wnt and Notch. High levels of BMP activity suppress anterior neural development, whereas abrogation of BMP signaling can promote neural specification (Wilson and Houart, 2004; Lamb et al., 1993; Bachiller et al., 2000). *In vitro* the addition of BMP2 and 4 to mouse neural stem cell cultures represses cell proliferation (Mathieu et al., 2008). BMP ligands regulate neuronal differentiation as well as determination of glial cell fate, by promoting astrocyte differentiation at the expense of oligodendrocytic fates (Mathieu et al., 2008; Yun et al., 2004; Sun and Xu, 2010; Gomes et al., 2003).

Radial glial cells (RGCs) begin to divide asymmetrically to start producing neurons at around 11.5 dpc in mice. RGC daughter cells detach from the ventricle and form the first neuronal layer of the preplate by 13.5 dpc (Haubensak et al., 2004). Subsequently born neurons migrate along RGCs and start to form the cortical plate, separating the preplate into Cajal-Retzius cells in the marginal zone and subplate neurons (Nichols and Olson, 2010; Marin-Padilla, 1978). The neocortex develops in an inside-out manner, with deep layers emerging first and superficial layers last. These neurons differentiate into glutamatergic pyramidal neurons, whereas inhibitory interneurons are born in the subcortical ganglionic eminences. Deep-layer pyramidal neurons (DLPNs, layer V and VI) have both intratelenchephalic projections to superficial cortical layers and extracortical projections to other brain regions (Saiki et al., 2017). Multiple molecular mechanisms regulate this corticogenesis (Kowalczyk et al., 2009; Franco et al., 2012; Chen et al., 2005); however, the role of BMP signaling in cortical layer formation and functional maturation of neurons has only been reported in limited studies so far. *In utero* electroporation of BMP7 to murine 14.5 dpc cortical ventricular cells impaired neuronal migration, suggesting that BMP signaling regulates neuronal positioning and migration (Choe and Pleasure, 2018). BMPs also regulate dendritogenesis and neurite growth *in vitro* (Lee-Hoeflich et al., 2004; Matsuura et al., 2007). This is consistent with a recent study that suggests perturbation of BMP signaling by delivery of a dominant-negative version of BMP IB receptor affects migration, polarity and dendritogenesis of mouse cortical neurons *in vivo* (Saxena et al., 2018).

To further establish the role of BMP signaling in forebrain development and neuronal function, we focus here on the expression and function of the BMP antagonist Grem1 in the developing mouse brain. We first assessed *Grem1* expression using transgenic *Grem1* reporter mice in the dorsal telencephalon and developing neocortex. Next, to investigate the role of *Grem1* in the developing brain, we used transcriptomic analyses of sorted mouse *Grem1*-expressing cells and single cell RNA-sequence (scRNA-seq) data, combined with mouse neural stem/progenitor cell (NSPC) culture *ex vivo*. Lastly, to examine the functional contribution of Grem1 to cortical development, we conditionally deleted *Grem1* in the dorsal telencephalon and undertook behavioral testing of mutant animals and littermate controls.

## RESULTS

### *Grem1*-expressing cells are located in the dorsal telencephalon and give rise to deep-layer neocortical neurons

**Fig. 1.**
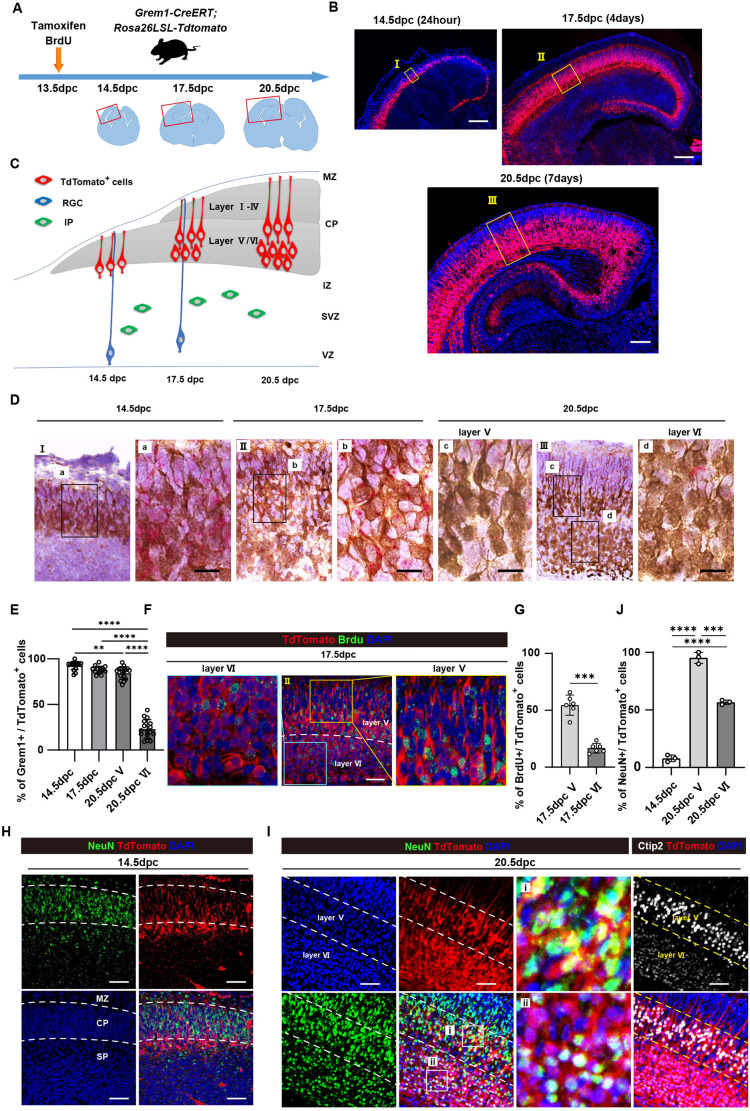
***Grem1*-expressing cells give rise to cortical neurons in the developing mouse brain.** (A) Schematic showing tamoxifen and BrdU administration to *Grem1creERT; Rosa26LSLTdTomato* (*Grem1*-reporter) mice. (B) Representative images of TdTomato^+^ (red) cells in the telencephalon shown with red boxes in A, at 14.5 dpc (24 h post-induction), 17.5 dpc (3 days post-induction) and 20.5 dpc (6 days post-induction) in *Grem1*-reporter mice treated with tamoxifen at 13.5 dpc, and DAPI staining (blue). (C) Schematic showing Tdtomato traces cells differentiating to layer V and VI neurons. (D) Representative images of immunohistochemical staining of boxed region from B to visualize TdTomato^+^ cells (red) and *Grem1* mRNA by ISH (green) at 14.5 dpc (I, 24 h post-induction), 17.5 dpc (II, 3 days post-induction) and 20.5 dpc (III, 6 days post-induction) in *Grem1*-reporter mice treated with tamoxifen at 13.5 dpc, and DAPI staining (blue). The boxed areas were further magnified in adjacent panels. (E) Quantification of D showing the percentage of TdTomato^+^ cells that were also *Grem1* RNA^+^ in four high-power fields (HPFs) of four biological replicates. One way ANOVA with Tukey's multiple test. (F) Representative images of immunofluorescence staining of 17.5 dpc telencephalon from *Grem1*-reporter (red) mice induced with tamoxifen at 13.5 dpc: BrdU, green; DAPI, blue. (G) Quantification of F showing the percentage of TdTomato^+^ cells that were also BrdU^+^ – two HPFs of three biological replicates, two-tailed, unpaired *t*-test. (H) Representative images of immunofluorescence staining of 14.5 neocortex from *Grem1*-reporter (red) mice induced with tamoxifen at 13.5 dpc: NeuN, green; DAPI, blue. MZ, marginal zone; CP, cortical plate; SP, subplate. (I) Representative images of immunofluorescence staining of 20.5 dpc neocortex from *Grem1*-reporter (red) mice induced with tamoxifen at 13.5 dpc: NeuN, green; DAPI, blue; Ctip2, white. Layer V and VI recognized with Ctip2 are boxed in (i) and (ii), respectively. (J) Quantification of H showing the percentage of TdTomato^+^ cells that were also NeuN^+^ in three HPF from three biological replicates. One way ANOVA with Tukey's multiple test. **P<0.01, ***P<0.001, ****P<0.0001. Data are mean±s.d. Scale bars: 200 µm (B); 20 µm (D); 50 µm (F,H,I).

*Grem1* RNA was first detected by *in situ* hybridization (ISH) in the mouse dorsal telencephalon at 13.5 dpc and expression was maintained through embryonic development until 20.5 dpc (Fig. S1A). To further assess *Grem1* expression in the embryonic mouse brain, we used transgenic *Grem1creERT; Rosa26LSLTdtomato* reporter mice, in which tamoxifen treatment results in expression of TdTomato in cells in which the *Grem1* enhancer and promoter sequences are active, and the progeny of those cells (Worthley et al., 2015). Pregnant *Grem1creERT; Rosa26LSLTdtomato* mice were administered tamoxifen at 11.5 dpc, the time point at which RGCs begin dividing in the developing cortex, and embryonic brains were collected 24 h later. Consistent with our *Grem1* ISH analysis, no TdTomato^+^ cells were observed, confirming that *Grem1* is not yet expressed in the brain at this early time-point (Fig. S1B). Next, we administered tamoxifen to pregnant dams at 13.5 dpc when *Grem1* is first expressed, and collected embryonic brains at 14.5, 17.5 and 20.5 dpc (Fig. 1A,B). From this time point we observed cells expressing TdTomato 24 h after tamoxifen administration located in the lower cortical plate and subplate of the dorsal telencephalon, with dendrites extending to the pia mater (Fig. 1B,C). ISH confirmed that *Grem1* RNA was detected in almost all TdTomato^+^ cells at 14.5 dpc, confirming that the reporter line recapitulates endogenous Grem1 expression (Fig. 1D,E). Six days later at 20.5 dpc, almost all layer V TdTomato^+^ cells still expressed *Grem1* RNA, whereas a significantly lower percentage of TdTomato^+^ layer VI neurons continued to express *Grem1* RNA (Fig. 1D,E). Further co-staining of TdTomato^+^ cells with cell type- and layer-specific markers showed that, at 20.5 dpc, the TdTomato^+^ cells have become Ctip2^+^ (Bcl11b^+^) layer V pyramidal neurons with dendrites extending to the pia mater and layer VI neurons (Fig. 1I; Fig. S1C). TdTomato^+^ cells did not express the layer II-IV marker, CDP (Cux1) (Fig. S1C). *Grem1* mRNA levels dramatically decreased after birth as determined by qRT-PCR analysis of total RNA isolated from mouse brain cortex at birth, postnatal day (P) 10 and 4 weeks post-birth (Fig. S1D).

To investigate when cells that become *Grem1^+^* are born during mouse cortical development, we administered the thymidine analogue bromodeoxyuridine (BrdU) concomitantly with tamoxifen to pregnant *Grem1creERT; Rosa26LSLTdtomato* dams at 13.5 dpc when it will be incorporated into dividing RGCs and intermediate progenitor cells (IPs) (Fig. 1A). We found that 55% of TdTomato^+^ layer V pyramidal neurons cells were labelled with BrdU at 17.5 dpc (Fig. 1F,G), demonstrating that these cells are derived from progenitors at embryonic day (E)13.5, with *Grem1* then expressed in committed layer V/VI neurons migrating over the following 3 days towards the cortical plate. Fewer TdTomato^+^ cells (17%) in layer VI were BrdU^+^ (Fig. 1F,G), consistent with layer VI neurons starting to be born before E13.5. At 14.5 dpc, TdTomato^+^ cells were also Ki67^−^ (Mki67^−^), suggesting that they were non-proliferative (Fig. S1E). We subsequently assessed whether TdTomato^+^ cells express a marker of post-mitotic mature neurons, NeuN (Rbfox3), by immunohistochemistry (IHC). At 14.5 dpc most TdTomato^+^ cells were NeuN^−^ and were located in the subplate, and lower NeuN^+^ cortical plate (Fig. 1H). In contrast, later in gestation at 20.5 dpc almost all TdTomato^+^ cells in Ctip2-strongly-positive layer V neurons and approximately half of Ctip2-weakly-positive layer VI neurons expressed NeuN (Fig. 1I,J). Together, these data suggest that *Grem1* starts being expressed by postmitotic cortical neurons destined for deep layers at E13.5 (Fig. 1C).

### Global transcriptomic analysis defines transcript modules enriched in *Grem1*-expressing cells

**Fig. 2.**
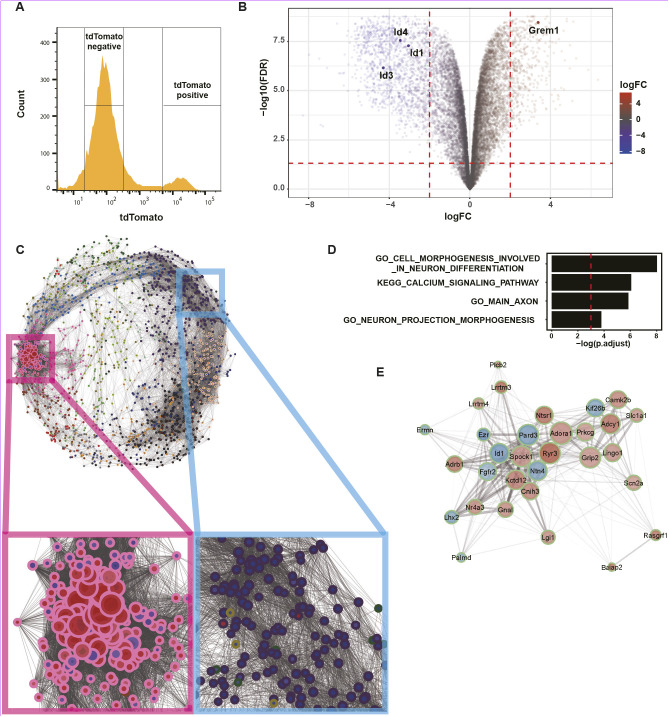
**Grem1-expressing cells have neuron-associated gene signatures.** (A) TdTomato^+^ and TdTomato^−^ cells were isolated from 14.5 dpc brains of *Grem1-CreERT; R26-TdTomato* mice induced with tamoxifen at 13.5 dpc. Representative FACS plot is shown, *n*=5 mice sorted for bulk RNA-seq analysis. (B) Volcano plot to show DEG between TdTomato^+^ and TdTomato^−^ cells from A. *Grem1* was significantly upregulated, whereas BMP target genes *Id1*/*3*/*4* were significantly downregulated in TdTomato^+^ cells compared with TdTomato^−^ cells. Absolute value of log2 fold change≥2.0, FDR<0.05. (C) Network visualization of correlated DEG modules in TdTomato^+^ cells. Each dot represents a gene, dot perimeter color indicates module membership (see Fig. S2), dot interior color denotes upregulation (red) or downregulation (blue) of gene transcript in TdTomato^+^ cells compared with TdTomato^−^, dot size indicates the magnitude of gene expression correlation to neighboring genes, lines connecting dots represent topological distance. The module containing highly correlated genes that were predominantly upregulated in TdTomato^+^ cells is boxed in magenta. (D) Significantly enriched gene sets in magenta boxed module in C. FDR<0.001. (E) Genes associated with *Id1*, a hub gene in the magenta boxed module in C. Dot perimeter color indicates module membership (see Fig. S2), dot interior color denotes upregulation (red) or downregulation (blue) of gene transcript in TdTomato^+^ cells compared with TdTomato^−^.

To further characterize *Grem1* expressing cells in the developing mouse brain, we undertook mRNA-seq analysis on TdTomato^+^ and TdTomato^−^ cells from the telencephalon. Pregnant *Grem1creERT; Rosa26LSLTdtomato* mice were administered tamoxifen at 13.5 dpc and embryonic brains were collected at 14.5 dpc. At this time point almost all TdTomato^+^ cells expressed endogenous *Grem1* RNA (Fig. 1D,E). Dissociated brain cells were flow sorted and live TdTomato^+^ and TdTomato^−^ cells were collected for transcriptomic analysis. TdTomato^+^ cells accounted for 5.1±2.1% of all live cells (*n*=5; mean±s.d.) (Fig. 2A). Bulk mRNA sequencing of TdTomato^+^ and TdTomato^−^ populations and analysis of 1845 differentially expressed genes (DEG) between the two cell populations revealed that *Grem1* was significantly upregulated in TdTomato^+^ cells and BMP transcriptional target genes (*Id1*/*3*/*4*) were significantly downregulated [adjusted *P*-value (FDR)≤0.05; Fig. 2B]. We next undertook a correlation analysis to identify modules of the 1845 DEGs that are coordinately regulated in TdTomato^+^ cells (Fig. S2). Shared membership of a module can suggest genes that together perform a particular function. The clustering tree depicts the topological distance between different modules, i.e. how similar or different the expression of transcripts within the module are from other modules, and highlighted one module that contained 288 genes with particularly tightly correlated transcripts (magenta, Fig. S2). Network visualization of the correlation analysis showed that the majority of transcripts within this tightly correlated module are upregulated in TdTomato^+^ cells (magenta square, Fig. 2C). We investigated the known function of genes within this module using a hypogeometric test for gene set enrichment. Significantly enriched pathways (adjusted *P* value (FDR) ≤0.001) were related to neuronal differentiation and projection, calcium signaling (possibly associated with synapse functions) and axons (Fig. 2D). The BMP target gene *Id1* was identified as one of the central hub genes in this cluster and its expression was significantly associated with other transcripts that have functions in neuronal maturation (such as Lrrtm3, Ryr3) (Fig. 2E). Consistent with our earlier identification of *Grem1*-expressing cells as committed neurons *in vivo*, this suggests that neuronal functions are upregulated in the *Grem1*-expressing TdTomato^+^ cells of the developing telencephalon.

### *Grem1/GREM1* is enriched in excitatory neuronal lineage cells during brain development

**Fig. 3.**
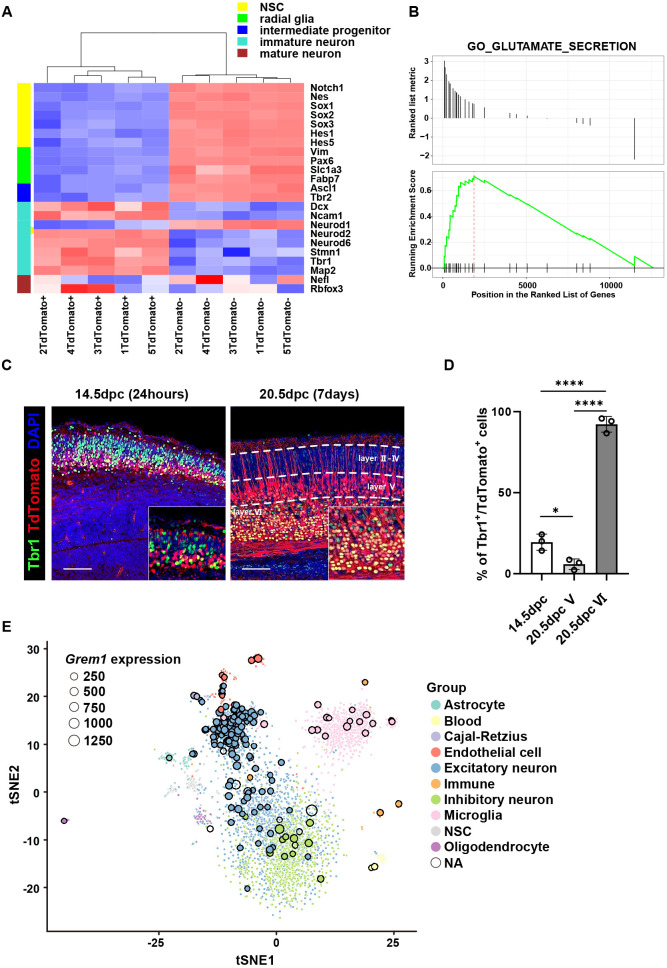
***Grem1* is expressed in the glutamatergic excitatory neuron lineage cells.** (A) Heatmap depicting unsupervised clustering of TdTomato^+^ and TdTomato^−^ cells isolated from 14.5 dpc *Grem1*-reporter (red) mice induced with tamoxifen at 13.5 dpc based on expression of representative differentiation marker transcripts for neural stem cell (NSC), radial glia, intermediate progenitor, immature neuron and mature neuron. (B) GSEA for Glutamate Secretion genes between TdTomato+ and TdTomato^−^ samples from A. NES=2.16, *P*=0.0049. (C) Representative images of immunofluorescence staining of 14.5 and 20.5 dpc telencephalon from *Grem1*-reporter (red) mice induced with tamoxifen at 13.5 dpc: Tbr1, green; DAPI, blue. (D) Quantification of C showing the percentage of TdTomato^+^ cells that were also Tbr1^+^ in three representative fields from three biological replicates. One way ANOVA with Tukey's multiple test. *P<0.05, ****P<0.0001. Data are mean±s.d. (E) tSNE plot of human scRNA-seq dataset. *GREM1*-expressing cells outlined in black. Dot size represents Grem1 expression value as indicated. NA, not applicable. Scale bars: 100 µm.

To further characterize the *Grem1-*expressing TdTomato^+^ cell population we undertook a candidate gene profiling approach using the bulk mRNA-seq data. Gene expression patterns of neuronal markers from a series of differentiation stages are shown in Fig. 3A. Immature neuronal markers, such as *Dcx*, *Ncam1*, *Neurod2*/*6* and *Tbr1*, were significantly upregulated at the RNA level in TdTomato^+^ cells in comparison with the TdTomato^−^ cells at 14.5 dpc, whereas neural stem cell, radial glia and intermediate progenitor transcripts were significantly under-represented (FDR<0.05, quasi-likelihood F-test). When RGCs (marked by co-expression of Sox2 and Pax6) generate neocortical neurons, the expression of specific transcription factors can be used to identify neurons within particular cortical layers (Franco et al., 2012; Chen et al., 2005). Our bulk RNA-seq analysis showed that transcripts encoding the layer V marker *Fezf2* and VI markers *Tbr1* and *Sox5* were significantly upregulated in *Grem1*-expressing TdTomato^+^ cells, whereas the RGC markers *Sox2* and *Pax6* and an intermediate progenitor marker, *Tbr2* (*Eomes*), were significantly downregulated (absolute value of log2 fold change≥2.0, FDR<0.05) and did not appear to colocalize with TdTomato at 14.5 dpc using IHC (Fig. S3).

To assist with identification of the neuronal subtype likely generated from *Grem1-*expressing TdTomato^+^ cells, we also performed Gene Set Enrichment Analysis (GSEA) on DEG transcripts between TdTomato^+^ and TdTomato^−^ cells. We found a significant enrichment of a glutamate secretion gene set in the DEGs [normalized enrichment score (NES)=2.16, *P*=0.0049] (Fig. 3B), whereas GABAergic and dopaminergic gene sets were not enriched (NES=−0.93, *P*=0.75; NES=−0.69, *P*=0.87, respectively) (Fig. S4A). Consistent with the GSEA, glutamatergic neuron markers, such as Slc17a7 (vGlut1), Grin1 and Grin2b were significantly upregulated in *Grem1*-expressing cells at 14.5 dpc using a candidate gene approach, whereas gabaergic and dopaminergic neuron markers, such as Slc6a1 (GABA transporter 1), Gad1, Gad2, and Th (tyrosine hydroxylase) were significantly downregulated (*P*<0.05, Fig. S4B). Tbr1 plays a central role in the development of early-born cortical excitatory neurons and regulates the connectivity of layer VI neurons (Hevner et al., 2001). To confirm that *Grem1*^+^ cells generate excitatory neuronal lineages, we undertook immunohistochemical staining with Tbr1. This revealed that *Grem1-*expressing TdTomato^+^ cells at 14.5 dpc expressed *Tbr1* at the mRNA level, but very low to no Tbr1 at the protein level; however, *Grem1-*TdTomato^+^ cells did become Tbr1^+^ neurons in cortical layer VI by 20.5 dpc (Fig. 3C,D).

To understand the relevance of our mouse focused study to the human setting, we reanalyzed publicly available human scRNA-seq data generated from human brain at mid-gestation (22-23 weeks post-conception) (Fan et al., 2018). We categorized each human cell in the dataset as either *GREM1^+^* or *GREM1^−^* based on the presence or absence of *GREM1* RNA-seq counts for each cell. Next, we compared the transcriptional profiles of our mouse embryonic brain *Grem1*-expressing TdTomato^+^ cells and TdTomato^−^ cells with the human mid-gestational brain *GREM1^+^* and *GREM1^−^* cells using a multidimensional scaling plot (Fig. S4C). Samples within the same group cluster tightly together, dimension 2 separates the samples along species lines, whereas dimension 1 clearly shows a similar separation of samples based on altered expression profiles depending on the *Grem1/GREM1* status in mouse and human developing brain (Fig. S4C). Mapping the *GREM1*^+^ cells onto a tSNE plot generated from the human scRNA-seq data revealed that most *GREM1-*expressing cells accumulated in the excitatory neuron cluster within the human mid-gestational cortex (Fig. 3E). Re-analysis of a mouse scRNA-seq cortical neuron dataset (Yuzwa et al., 2017) also confirmed that expression of *Grem1* most highly correlates with the excitatory neuron cluster marked by *Foxp2* (marker of layer VI) and *Ctip2* (marker of layer V/VI) and not precursor or progenitor populations (Fig. S5D). Lastly, we analyzed the expression of other BMP-signaling components in both scRNA-seq datasets (Fig. S5A-D). Of the pathway antagonists similar to *GREM1*, sclerostin domain-containing protein 1 (*SOSTDC1*), *CHRD*, BMP binding endothelial regulator (*BMPER*) and follistatin (*FST*) transcripts were highly enriched in the excitatory neuron or Cajal-Retzius cell clusters, whereas *NOG* expression correlated only with the astrocyte population in the human data that contained a broad selection of neuronal and non-neuronal cell types (Fig. S5A). This analysis of BMP antagonist expression was further extended by the increased resolution of neuronal cell clusters found in the multi-time point and neuronal-restricted mouse scRNA-seq dataset, to be enriched in excitatory neurons and apical precursors/RGCs (Fig. S5D). BMP2/7 that are antagonized by GREM1 were primarily produced by alternate RGCs and IPs/excitatory neuron populations to the *Grem1-*expressing excitatory neuron population in the mouse developing cortex (Fig. S5D). Expression of *BMP2*/*7* was also inversely correlated with *GREM1* expression in the human excitatory neuron cell population, suggesting paracrine BMP pathway regulation by these opposing factors (Fig. S5C). Expression of the BMP target genes, *ID1*/*3*/*4*, was inversely correlated with *GREM1*-expression in excitatory neurons, consistent with our developmental mouse brain data and suggesting that *GREM1* acts locally to antagonize BMP signaling (Fig. 2B). BMP receptor type I and II expression correlated with apical precursor and proliferative RGC populations (*Bmpr1a*/*1b*, *Acvr2b*) and excitatory neuron clusters in the mouse scRNA-seq dataset (*Alk*, *Acvr1b*/*1*/*1c*/*2a*, *Bmpr2*), suggesting that many of the neurons in the developing cortex will have the ability to respond to BMP pathway stimulation (Fig. S5A,D). This highlights the complexity of regulation of BMP signaling during development of the cortex.

### Grem1 promotes proliferation and neural differentiation in NSPCs *ex vivo*

**Fig. 4.**
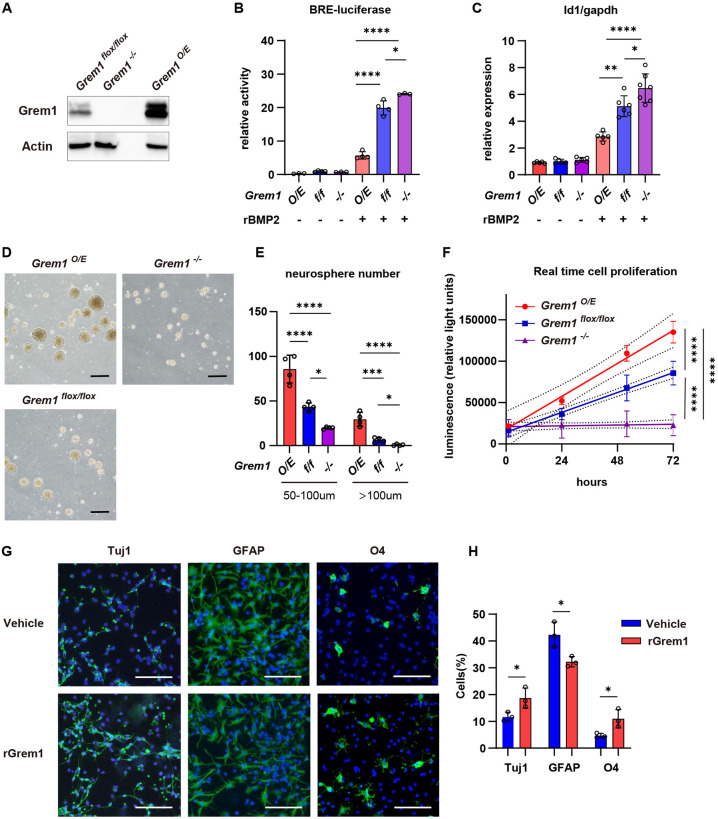
**Grem1 promotes proliferation and neuronal differentiation in neural stem/progenitor cells *ex vivo.*** (A) Detection of Grem1 protein levels in control *Grem1^flox/flox^*, *Grem1^−/−^* and *Grem1^O/E^* NSPC by western blot. (B) BRE-reporter activity relative to internal control reporter. Data obtained from four independent experiments each performed in triplicate. One way ANOVA with Tukey's multiple test. (C) BMP target gene *Id1* mRNA expression normalized to *Gapdh* in *Grem1^flox/flox^*, *Grem1^−/−^* and *Grem1^O/E^* NSPC treated with vehicle or rBMP2 for 24 h. One way ANOVA with Tukey's multiple test. Results from five independent experiments performed in triplicate. (D) Representative images of *Grem1^flox/flox^*, *Grem1^−/−^* and *Grem1^O/E^* neurosphere cultures. (E) Quantification of neurosphere number and size from D in four representative fields from four independent experiments. One way ANOVA with Tukey's multiple test. (F) Cell proliferation normalized to 0 h time point for each culture. Results from three independent experiments each performed in triplicate and analyzed using linear regression. (G) Representative images of immunofluorescence staining to identify Tuj1^+^ neurons, GFAP^+^ astrocytes and O4^+^ oligodendrocytes in differentiated cultures from wild-type NSPCs treated with vehicle or rGrem1. (H) Quantification of G, showing the percentage of marker^+^ cells in three representative fields from three independent experiments. Two-tailed, unpaired *t*-test. Data are mean±s.d. *P<0.05, **P<0.01, ***P<0.001, ****P<0.0001. Scale bars: 100 µm.

Next we collected NSPCs from embryonic brains of *Grem1^flox/flox^* mice at 14.5 dpc and transduced the cells *ex vivo* with control, *Cre*-expressing or *Grem1*-expressing lentivirus to generate control *Grem1^flox/flox^*, *Grem1^−/−^* and *Grem1* overexpressing (*Grem1^O/E^*) primary cultures. Endogenous Grem1 was detected in *Grem1^flox/flox^* control cultures by western blot, with absent or elevated protein levels in *Grem1^−/−^* and *Grem1^O/E^* cultures, respectively (Fig. 4A). There were two bands, likely because of splicing variants, consistent with previous reports (Yan et al., 2014; Koli et al., 2016). Our method does not include selection for NSPC markers or exclusion of committed lineage cells and so contains a heterogeneous mix of cells that are roughly selected for proliferation potential. Because of this, it is possible that some of this mixed population may include immature committed neurons that express Grem1, or Grem1 expression may be aberrantly upregulated in stem/progenitor cells by culture *in vitro*. Control *Grem1^flox/flox^* cultures were responsive to BMP pathway induction as determined by increased BRE-luciferase reporter activity and expression of the BMP target genes *Id1*/*2*/*3*/*4* following addition of recombinant human BMP2 (Fig. 4B,C; Fig. S6). This induction of BRE-reporter activity and increase in BMP target gene transcript levels was significantly enhanced in *Grem1^−/−^* cells and attenuated in *Grem1^O/E^* cells (Fig. 4B,C; Fig. S6). This confirmed that Grem1 acts as an antagonist of BMP2 and suppresses downstream transcriptional targets in embryonic NSPCs. This difference was more obvious with *Grem1* overexpression than genetic deletion, possibly due to limited basal expression of Grem1 in NSPC culture.

We next employed neurosphere assays to determine the effect of Grem1 modulation on proliferation of embryonic NSPCs. Only mitogen-responsive cells proliferate to form clusters termed neurospheres, where sphere size correlates with proliferative ability (Mori et al., 2006; Reynolds and Rietze, 2005). *Grem1^O/E^* cells form significantly more and larger neurospheres than control *Grem1^flox/flox^* cells, whereas *Grem1^−/−^* cells form significantly fewer (Fig. 4D,E). To understand this phenomenon from the aspect of cell viability, we evaluated the proliferation rates of *Grem1^flox/flox^*, *Grem1^−/−^* and *Grem1^O/E^* cultures. Overexpression of *Grem1* significantly increased the rate of NSPC proliferation compared with control *Grem1^flox/flox^* NSPCs, whereas the number of viable *Grem1^−/−^* cells did not increase over the 72 h time course analyzed (Fig. 4F). Our re-analysis of the mouse neuronal cortex scRNA-seq dataset (GSE107122) showed that BMP2/7, which are antagonized by GREM1, were primarily produced by RGCs and IPs/excitatory neurons in the developing mouse cortex (Fig. S5D), suggesting BMP2/7 may be produced by the NSPCs in our *in vitro* system. These results suggested that Grem1 contributes to the proliferation of NSPCs. Following on from this observation, we wanted to assess the role of Grem1 in the differentiation of NSPCs to neurons, astrocytes and oligodendrocytes. When cultured in recombinant BMP2-containing differentiation media, addition of 1 ng/ml recombinant GREM1 significantly increased the number of NSPCs that differentiated into Tuj1^+^ (Tubb3^+^) neurons and O4^+^ (Foxo4^+^) oligodendrocyte lineage cells, and decreased the number of GFAP^+^ astrocytes, in comparison with vehicle treated control NPSCs (Fig. 4G,H). This suggests that Grem1 may regulate the differentiation potential of NPSCs.

### *Grem1* is required for normal cortical development

**Fig. 5.**
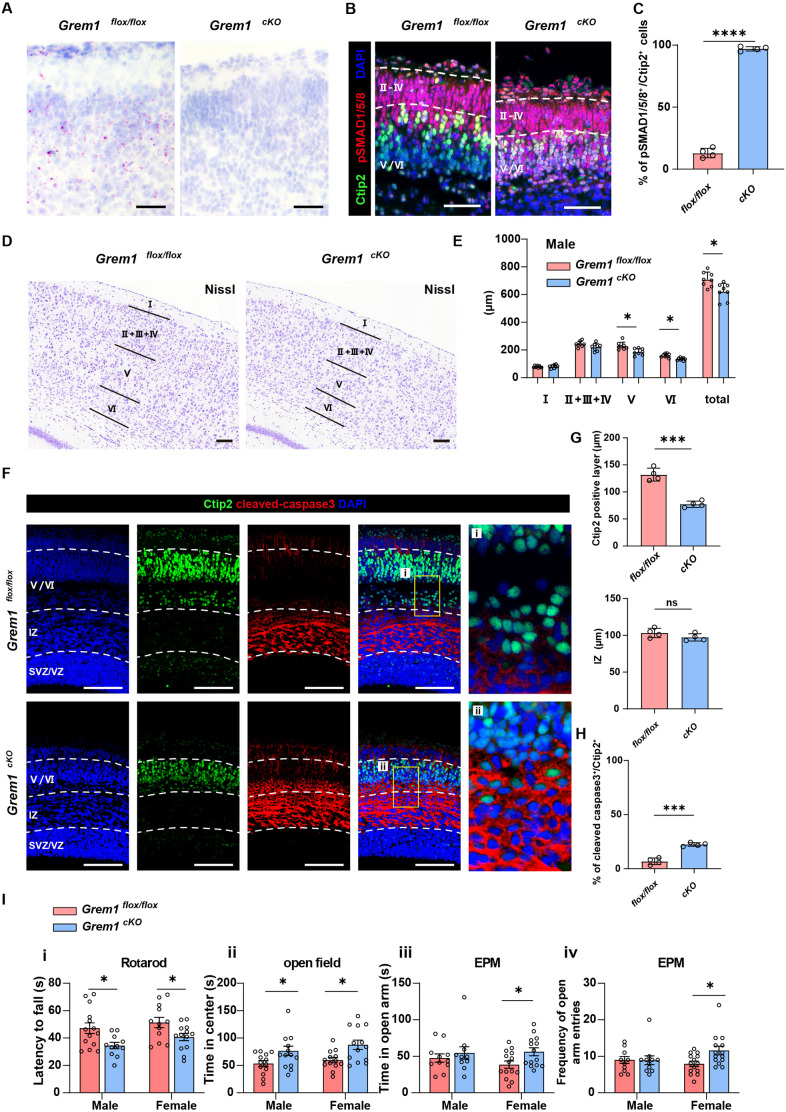
**Cortical development, motor balance and fear is impaired in *Grem1* conditional knockout mice: *Grem1* is required for normal cortical development.** (A) Representative *Grem1* expression in the dorsal telencephalon of *Grem1^cKO^* mice and *Grem1^flox/flox^* littermate controls by ISH at 20.5 dpc. *n*=3. (B) Representative images of immunofluorescence staining of cortex of *Grem1^cKO^* mice and littermate controls at 20.5 dpc with layer V and VI showing marker Ctip2 (green), phosphorylated SMAD1/5/8 (red) and DAPI (blue) (see also Fig. S7C). (C) Quantification of phosphorylated SMAD1/5/8^+^ cells in Ctip2^+^ cells in (B). *n*=4, two-tailed, unpaired *t*-test. (D) Representative histological images of cortical layers of *Grem1^cKO^* mice and *Grem1^flox/flox^* littermate controls using Nissl staining at 10 weeks of age. (E) Quantification of cortical layer thickness from D compared in eight pairs of male *Grem1^cKO^* mice and littermate controls at 10 weeks of age. Male, *n*=7 control and *n*=7 *Grem1^cKO^*, two-tailed, unpaired *t*-test. (F) Representative images of immunofluorescence staining of cortex of *Grem1^cKO^* mice and littermate controls at 20.5 dpc with layer V and VI showing marker Ctip2 (green), cleaved caspase 3 (red) and DAPI (blue). *n*=4. High magnification of boxed areas is shown in right panels. (G) Quantification of Ctip2^+^ layer V/VI and intermediate zone thickness from F compared in four pairs of *Grem1^cKO^* mice and littermate controls at 20.5 dpc, two-tailed, unpaired *t*-test. (H) Quantification of cleaved caspase 3^+^ cells in Ctip2^+^ cells in G. *n*=4, two-tailed, unpaired *t*-test. (I) Behavioral testing was performed to compare *Grem1^cKO^* and *Grem1^flox/flox^* mice using age and sex matched littermates at 7-10 weeks of age. (i) Rotarod test latency to fall. Male, *n*=14 control and *n*=11 *Grem1^cKO^*; Female, *n*=12 control and *n*=13 *Grem1^cKO^*, two-tailed, unpaired *t*-test. (ii) Open field test, cumulative duration spent in the center area. Male, *n*=13 control and *n*=14 *Grem1^cKO^*; Female, *n*=13 control and *n*=13 *Grem1^cKO^*, two-tailed, unpaired *t*-test. (iii,iv) Elevated plus maze test. (iii) Cumulative duration spent in open arms. (iv) The number of entries to open arms. Male, *n*=12 control and *n*=11 *Grem1^cKO^*; Female, *n*=14 control and *n*=14 *Grem1^cKO^*, two-tailed, unpaired *t*-test. Data are mean±s.e.m. *P<0.05, ***P<0.001, ****P<0.0001. Scale bars: 100 µm.

In order to assess the functional role of *Grem1* in mouse forebrain development *in vivo*, we generated tissue-specific *Grem1* conditional knockout mice using the empty spiracles homeobox 1 (*Emx1*)-*cre* driver. We first verified that *Emx1-cre* generates efficient cre-mediated recombination in the dorsal telencephalon at 14.5 dpc by visualizing TdTomato^+^ cells in reporter *Emx1-cre; Rosa26LSLTdtomato* mouse brains (Fig. S7A). Next we confirmed that *Emx1-cre; Grem1^flox/flox^* (*Grem1* conditional knockout, *Grem1^cKO^*) mice had a significant reduction in *Grem1* RNA in the developing forebrain compared with cre^−^ littermate controls by ISH at 14.5 dpc (Fig. 5A) and real time RT-PCR at P0 (Fig. S7B). *Grem1^cKO^* mice were viable and fertile. To determine the mechanistic effect of loss of *Grem1* we directly analyzed BMP pathway activity by immunohistochemical staining for the downstream pathway effectors, phospho-SMAD1/5/8. The number of Ctip2^+^ layer V/VI cells with active BMP signaling (phospho-SMAD1/5/8^+^) was significantly increased in *Grem1^cKO^* mice in comparison with *Grem1^flox/flox^* littermate controls (Fig. 5B,C; Fig. S7C). To examine the morphological consequences of conditional *Grem1* loss in the developing mouse brain we performed Nissl staining on tissue samples from *Grem1^cKO^* and cre^−^ littermate controls. At 10 weeks of age, total cortical thickness was significantly reduced in *Grem1^cKO^* mice in comparison with *Grem1^flox/flox^* littermate controls both in males and females, owing to significantly thinner cortical layers V and VI as marked by Ctip2 and Foxp2 (Fig. 5D,E; Fig. S7D,E,F). Cellular density was also lower in *Grem1^cKO^* mice in comparison with *Grem1^flox/flox^* littermate controls (Fig. S7G,H). The number of Ki67^+^ proliferative cells was unchanged both at 14.5 dpc and 20.5 dpc between *Grem1^cKO^* and *Grem1^flox/flox^* littermate controls (Fig. S7I,J,K,L). Also the number of Tbr2^+^ cells was unchanged at 14.5 dpc (Fig. S7I,J). In contrast, the number of cleaved caspase 3^+^ cells was increased in Ctip2^+^ layers of *Grem1^cKO^* mice in comparison with *Grem1^flox/flox^* littermate controls at 20.5 dpc, but was not different at 14.5 dpc (Fig. 5F,H; Fig. S7M). This increase in apoptotic cells marked by cleaved caspase 3 likely explains the significantly thinner Ctip2^+^ layer we observed in *Grem1^cKO^* animals compared with *Grem1^flox/flox^* littermate controls; however, our analyses have not ruled out potential changes in cell proliferation or cell fate decisions between the 14.5 and 20.5 dpc time points examined (Fig. 5G). Conversely, the other predominant region of *Emx1-cre* driver activity, the hippocampus, which has important functions in memory for navigation pertinent to our behavioral testing, displayed morphology and cell density that did not appear to be different to normal on microscopic inspection (Fig. S8A).

Our earlier transcriptional network analysis from bulk RNA-seq data identified an *Id1*-associated gene cluster that was differentially regulated in the *Grem1-*expressing TdTomato^+^ cells from the cortex at 14.5 dpc (Fig. 2E). From this cluster, *Lrrtm3* and *Ryr3* were selected for further analysis because of their known functions in synapse development (Balschun et al., 1999; Um et al., 2016; Roppongi et al., 2017; Futagi and Kitano, 2015; Linhoff et al., 2009). The expression of these two transcripts was significantly downregulated in the cortex of *Grem1^cKO^* mice compared with littermate controls at P10 (Fig. S8B). This provides further evidence of the relationship between *Grem1* expression and these genes. To examine the effect of Grem1 deletion on differentiation, transcript levels of *Slc17a7* for excitatory neurons, *Gfap* for astrocytes and myelin-associated glycoprotein (*Mag*) for oligodendrocytes were also assessed by qPCR. Although *Slc17a7* was significantly decreased in *Grem1^cKO^* samples, there was no significant difference in *Gfap* or *Mag* (Fig. S8B).

### Motor coordination and fear responses were impaired in *Grem1^cKO^* mice

To assess the functional consequences of embryonic *Grem1* deletion in the mouse forebrain, we undertook behavioral testing with *Grem1^cKO^* mice in comparison with *Grem1^flox/flox^* littermate controls. We compared motor balance using the Rotarod test. Latency to fall was significantly shorter in *Grem1^cKO^* mice both for males and females, suggesting an impaired motor balance following loss of *Grem1* (Fig. 5I). During the open field test used to assess exploratory behaviors, both male and female *Grem1^cKO^* mice spent significantly more time in the central area away from the walls than littermate controls (Fig. 5I). The total distance moved and velocity of movement were similar between *Grem1^cKO^* and littermate controls (Fig. S8C,D). Behavioral testing using an elevated plus maze also indicated that female *Grem1^cKO^* mice spent significantly more time in the open maze arms and entered into the open arms more frequently than littermate controls (Fig. 5I). Both of the exploratory behavior tests indicated that conditional loss of *Grem1* leads to reduced anxiety-like behavior. Lastly, we used the Y maze test to assess the short-term memory of *Grem1^cKO^* and littermate controls. We observed no difference in the number of entries made to the novel arm of the Y maze between groups (Fig. S8E,F), suggesting that *Grem1* expression is not required for short-term memory function.

## DISCUSSION

The spatiotemporal regulation of BMP signaling in brain development is poorly understood. Here, we focus on the expression and function of the BMP antagonist Grem1 during brain development, using transgenic lineage tracing, gene expression, *in vitro* culture and conditional knockout approaches.

In the developing mouse brain *Grem1* is first expressed at 13.5 dpc in committed neurons that will differentiate into layer V and VI neurons by 20.5 dpc (Fig. 1I; Fig. S1A,C). Transcriptomic profiling using our mRNA-seq data from embryonic *Grem1*-reporter mouse brains and publicly available mouse and human developmental brain scRNA-seq data suggests that *Grem1*/*GREM1* expression is primarily associated with markers of glutamatergic excitatory neuronal lineages, rather than GABAergic or dopaminergic neurons or other cell types of the developing brain (Fig. 3B,E; Fig. S4A,B). Consistent with this, *Grem1* was not expressed in the ganglionic eminences, from which GABAergic interneurons originate; nor were GABAergic transcripts upregulated or enriched by GSEA in *Grem1*-expressing TdTomato^+^ cells in the dorsal telencephalon using bulk RNA-seq analysis (Fig. S4A,B). Likewise Grem1-lineage-traced cortical neurons expressed the excitatory marker Tbr1 (Fig. 3C). *Grem1*-expression labels and generates important excitatory neuronal lineages, i.e. pyramidal neurons, which play high-level cognitive functions in the neocortex. Grem1 expression is required for the correct development of early-born excitatory neurons of the neocortex (Figs 1I and 5D-H). Although we have not directly analyzed the proportion of excitatory and inhibitory neurons in *Grem1^cKO^* animals here, misregulation of BMP signaling might affect the balance between excitatory and inhibitory neuronal activities, which has been implicated in neurodevelopmental disorders (Dani et al., 2005).

Our analysis of scRNA-seq data from human midgestational cortex (22-23 weeks post-conception) and from mouse embryonic cortex suggests that there is coordinated regulation of BMP signaling during development, but that there may be also be functional redundancies (Fig. S5A,D). Nog is the most extensively studied of the BMP antagonists in the CNS, in which it is a recognized neural inducer during gastrulation (Lamb et al., 1993). Addition of recombinant Nog to neural stem cells promotes neuronal and suppresses astrocytic differentiation *in vitro* (Mikawa and Sato, 2011). In our study, we confirmed that Grem1 acts as a BMP antagonist in embryonic NSPC cultures, suppressing downstream BMP transcriptional targets (Fig. 4B,C; Figs S5C, S6). Although Grem1 is expressed by immature but committed neurons, rather than Sox2^+^ or Pax6^+^ neural stem or precursor populations *in vivo* (Fig. S3), this *in vitro* study was useful to simulate the role of secreted Grem1. Similar to Nog, we determined that Grem1 promotes proliferation and neural differentiation, at the expense of astrocytic lineages *in vitro* (Fig. 4G,H). This is consistent with a previous report that found Grem1 promotes proliferation and suppresses astrocyte differentiation of cancer stem cells in glioblastoma (Yan et al., 2014). *Grem1^cKO^* mice showed decreased Slc17a7 mRNA expression, but no significant change in glial cell differentiation, possibly due to the redundant BMP regulatory effects by other antagonists *in vivo*.

Our histological analyses identified that the cortex (particularly neocortical layers V and VI) of *Grem1^cKO^* animals is significantly thinner, with less cells, in comparison with littermate controls, with no obvious morphological changes in the hippocampus. In contrast to our NSPC *in vitro* data (Fig. 4D-F), we did not observe a significant decrease in the number of Ki67^+^ proliferative cells or Tbr2^+^ intermediate progenitors in *Grem1^cKO^* animals at 14.5 dpc or 20.5 dpc (Fig. S7I-L). However, it is possible that progenitors may have altered proliferation or differentiation in *Grem1^cKO^* animals between the time points analyzed. Still, the number of cleaved caspase 3^+^ apoptotic cells was increased in cortical layers V/VI in the absence of *Grem1* in *Grem1^cKO^* animals at 20.5 dpc, whereas Ctip2 and cleaved caspase 3 were not changed at 14.5 dpc (Fig. 5F,H; Fig. S7M). This suggests that apoptosis was increased in *Grem1^cKO^* after 14.5 dpc, which could contribute to the thinner deep cortical layers in these animals after birth in comparison with littermate controls.

Although secreted Grem1 can influence the upper as well as deep-layer neurons, we did not observe any noticeable changes to downstream BMP pathway activity in the upper layer neurons of *Grem1^cKO^* mice in comparison with *Grem1^flox/flox^* littermate controls as indicated by phospho-SMAD1/5/8 staining (Fig. 5B,C; Fig. S7C). We did however observe an increase in phospho-SMAD1/5/8 staining in Ctip2^+^ deep-layer neurons in *Grem1^cKO^* mice in comparison with *Grem1^flox/flox^* littermate controls. BMP transcriptional targets were significantly downregulated in Grem1-expressing TdTomato^+^ cells *in vivo* (Fig. 2B) and Grem1^+^ excitatory neurons in the human embryonic cortex (Fig. S5C). An upper layer neuron marker, *Cux1*, and expression of BMP receptors *Bmpr1a*/*1b* and *Avcr2b* correlated with apical precursor and proliferative RGC populations, whereas deep-layer neuron markers *Foxp2* and *Ctip2*, and BMP receptors *Alk*, *Acvr1b*/*1*/*1c*/*2a* and *Bmpr2* expression correlated with excitatory neuron or newborn neuron clusters in the mouse scRNA-seq cortical neuron dataset (Yuzwa et al., 2017) (Fig. S5D). This suggests that BMP receptors are expressed both in upper and deeper layer cortical neurons, although with specific cell-type expression patterns for each receptor. Conversely, BMPs are produced by endothelial cells, astrocytes and excitatory neurons in human (Fig.S5B). In the mouse cortex, BMPs are produced by RGCs and IPs/excitatory neuron populations, according to single cell analysis of neuronal lineage cells (Fig. S5D), and also from the meninges (Choe et al., 2012) (Fig. S5E). Although Grem1 is secreted, it usually acts on nearby or adjacent cells (Worthley et al., 2015). This could explain why downstream SMAD signaling is most affected in deep-layer neurons in which Grem1 is predominantly expressed. Together these results suggest that Grem1-expressing cells are fated to become neurons via antagonism of potential paracrine BMP signals (Fig. S5E) (Furuta et al., 1997; Imura et al., 2008; Choe and Pleasure, 2018).

Layer V neurons target the spinal cord, cerebellum, striatum and the thalamus (Shipp, 2007) and play roles in movement preparation, movement guidance and the execution of well-timed movements (Baker et al., 2018; Li et al., 2015). The observed decrease in layer V neurons would lead to impaired preparation for and coordination of movement in *Grem1^cKO^* animals and explain the motor balance defect in these animals in comparison with litter mate controls. Emerging evidence suggests that layer VI neurons play a central role in modulating thalamic and cortical neurons during sensory processing (Wang et al., 2016). Our behavioral testing confirmed that loss of Grem1 expression significantly impaired fear sensitivity in *Grem1^cKO^* animals, compared with littermate controls, consistent with a deficiency in layer VI neurons with roles in sensory connections. BMP signaling is known to promote synaptogenesis (Xiao et al., 2013; Shen et al., 2004) and altered synaptogenesis can affect both fear sensitivity and motor abilities (Ha et al., 2016; Wood and Shepherd, 2010). Network analysis of DEG between *Grem1*-expressing TdTomato^+^ and TdTomato^−^ cells from the developing mouse dorsal telencephalon identified a gene cluster of inter-related transcripts in which the BMP target gene, *Id1*, acts as a hub. Genes in the cluster play roles in neuron morphogenesis, synapse and axon maturation (Fig. 2D,E). For example, *Lrrtm3*/*4* encode synapse organizing proteins crucial for the development and function of excitatory synapses (Um et al., 2016; Roppongi et al., 2017; Linhoff et al., 2009). Likewise, *Ryr3* encodes a member of a family of receptors that shape synaptic transmissions(Balschun et al., 1999) by amplifying spike-driven calcium signals in presynaptic terminals, and consequently enhancing the efficacy of transmitter release (Futagi and Kitano, 2015). In *Grem1^cKO^* animals, the mRNA expression of *Lrrtm3* and *Ryr3* was significantly decreased in comparison with littermate controls (Fig. S8B). This is possibly because of a loss of excitatory deep-layer neurons in the gross cortex tissue samples from *Grem1^cKO^* animals compared with *Grem1^flox/flox^* littermate controls, rather than indicating a direct role for Grem1 in the regulation of synaptogenesis. However the potential role for Grem1 and other BMP modulators in neuron morphogenesis, synapse and axon maturation in the developing cortex warrants further interrogation.

In summary, this is the first study to reveal the important function of Grem1 in cortical development. Grem1 is expressed and essential for survival of committed deep-layer cortical neurons. In the future, Grem1 may hold value beyond understanding the cellular biology of brain development and function as we develop new approaches to help tackle complex neurodevelopmental and neurological diseases.

## MATERIALS AND METHODS

### Mice

*Grem1-CreERT* transgenic mice (Worthley et al., 2015) were crossed with *R26-LSLTdTomato* mice [B6.Cg-*Gt(ROSA)26Sor^tm14(CAG−tdTomato)Hze^*/J; JAX 007914] to generate a tamoxifen-induced *Grem1* reporter line. Pregnant dams were administered 6 mg of tamoxifen by oral gavage to induce embryonic *Grem1*-tracing, with some dams bearing 13.5 dpc embryos also intraperitoneally injected with BrdU (Roche, 40 mg/kg) to track cell division. *Grem1^flox/flox^* mice (Gazzerro et al., 2007) were crossed with *Emx1-cre* mice (B6.129S2-*Emx1^tm1(cre)Krj^*/J; JAX 005628) to generate *Emx1-cre*-mediated *Grem1* conditional knockout mice (*Emx1-cKO*). The line was maintained by crossing *Emx1*^*cre/+*^*; Grem1*^*flox/flox*^ mice with *Grem1^flox/flox^* mice.

All mice were on the C57BL6/J background and experimentation was conducted following approval by South Australian Health and Medical Research Institute (SAHMRI) Animal Ethics Committee (approval number SAM284) in accordance with the *Australian Code for the Care and Use of Animals for Scientific Purposes* (8th edition).

### Preparation of single cell suspensions and flow cytometry

Pregnant *Grem1creERT; Rosa26LSLTdTomato* mice were administered tamoxifen at 13.5 dpc. At 14.5 dpc, dorsal telencephalons of the embryos were dissected in cold phosphate-buffered saline (PBS) and the meningeal membranes were removed. Neurocult Enzymatic Dissociation kit (Stemcell Technologies) was used for cell dissociation according to the manufacturer's protocol. Dissociated cells were resuspended in fluorescence-activated cell sorting (FACS) buffer containing DAPI (0.5 µg/ml). Sorting and analyses were carried out on a FACS Fusion flow cytometer (Becton-Dickinson). Dead cells were excluded by gating on forward and side scatter and by eliminating DAPI^+^ events. The cells harvested from cre^−^ littermate control mice were used to set background fluorescence levels. Viable cells were sorted into FACS buffer, collected via centrifugation (300 ***g***) and resuspended in Trizol (Invitrogen) for RNA extraction.

### RNA extraction and mRNA-seq

RNA was extracted from sorted cells in Trizol according to the manufacturer's protocol with the exception of addition of glycogen (20 µg/µl) and isopropanol precipitation overnight at −80°C to maximize yield. RNA quality and quantity were analyzed using a NanoDrop and TapeStation (Thermo Scientific). Total RNA was converted to strand-specific Illumina-compatible sequencing libraries using the Nugen Universal Plus mRNA-Seq library kit from Tecan (Mannedorf) as per the manufacturer's instructions (MO1442 v2). Briefly, 500 ng of total RNA was polyA selected and the mRNA fragmented before reverse transcription and second strand cDNA synthesis using dUTP. The resultant cDNA was end repaired before the ligation of Illumina-compatible barcoded sequencing adapters. The cDNA libraries were strand selected and PCR amplified for 12 cycles before assessment by Agilent Tapestation for quality and Qubit fluorescence assay for quantity. Sequencing pools were generated by mixing equimolar amounts of compatible sample libraries based on the Qubit measurements. Sequencing of the library pool was performed with an Illumina Nextseq 500 using single read 75 bp (v2.0) sequencing chemistry.

### Bioinformatic analysis

#### RNA-seq data processing

Fastq files from the sequencing run were subjected to quality controls with FastQC version 0.11.3. Raw reads with low quality were removed using Trim Galore alignment (Phred score less than 28 and/or reads contains adaptor sequences). After trimming, all bases with low quality scores and adaptor sequences were removed. The trimmed reads were mapped to Ensembl mouse genome (GRCm38) with STAR 2.4.2a. No more than one base mismatch was allowed. Only uniquely mapped reads were retained. Option -quantMode was enabled to generate gene level quantification. The counts files were then merged into an expression table for downstream differential expression analysis.

#### Differential expression analysis

DEG between the *TdTomato*^+^ (*Grem1*^+^) and *TdTomato*^−^ (*Grem1*^−^) populations were analyzed with edgeR packages in R version 3.6.0 (Robinson et al., 2010). Reads were inspected through a multidimensional scaling plot and outliers were removed. Weighted trimmed mean of M values (TMM) normalized counts were log2-transformed and counts per million (CPM) obtained. Paired comparisons between the *TdTomato*^+^ (*Grem1*^+^) and *TdTomato*^−^ (*Grem1*^−^) populations were performed. DEG reported as significant were selected by requiring both adjusted *P*-value (FDR) ≤0.05 and absolute value of log2 fold change ≥2.0.

#### Supervised Weighted Gene Correlation Network Analysis (WGCNA)

Only DEGs were used for subsequent network analysis. The gene network was constructed using the R package supervised weighted gene correlation network analysis (WGCNA) following the procedure previously described (Langfelder and Horvath, 2008). After the low expression genes (FPKM<1) had been filtered out from all gene expression libraries, Pearson's correlation-based adjacency was calculated on the basis of pairwise correlations of gene expression within *TdTomato*^+^ (*Grem1*^+^) samples. Topological overlap of the correlations was used to weight the edges of the correlation network. The higher the weight, the stronger the interaction between two genes. Connectivity for a single gene was calculated as the sum of weights relative to the rest of the genes, and the top 5% of genes with the highest connectivity in the network defined as hub genes. For visualization, a heat map was generated using the TOMplot() function in WGCNA R package (version 1.68), with dissimilarity topological overlap (1– topological overlap), employed for hierarchical clustering. To generate the network plot, the weights of the network were cut off at 0.1. Hypergeometric enrichment tests for each module-defined gene list were performed with the enricher() function, as part of clusterProfiler R package version 3.13.0 (Yu et al., 2012). Bonferroni adjustment for *P*-value was used, only gene sets with an adjusted *P*-value <0.05 were considered for interpretation of the biological function modules.

#### Gene set enrichment analysis

To use the Molecular signatures database (MSigDB), we accessed all gene sets (.gmt file, version 6.2) from the Broad Institute, MA, USA, and chose a subset for further analyses including BioCarta, Hallmark, Gene Ontology (GO), Kyoto Encyclopedia of Genes and Genomes (KEGG), Pathway Interaction Database and the Reactome Pathway Database. To enable cross species comparisons, mouse gene ensembl IDs were converted to human orthologous gene symbols using biomaRt R package, version 2.41.7 (Durinck et al., 2009). Humanized gene lists for the whole transcriptome were ranked based on logfc values by comparing TdTomato^+^ and TdTomato^−^ cells. The R package clusterProfiler version 3.13.0 (Yu et al., 2012) was used to scan through all the gene sets mentioned above, using Benjamini–Hochberg adjusted *P*-values.

#### Analysis of publicly available scRNA-seq dataset

scRNA-seq raw expression data was accessed for human mid-gestational brain (22 and 23 weeks post-conception) cortex samples from GSE103723 (Fan et al., 2018). The cells from two individual samples were grouped together, with the sum of reads calculated for each gene to represent the data structure of bulky transcriptomics. The limma package (Ritchie et al., 2015) was used to remove batch effects with removeBatchEffect() and to generate multidimensional scaling plots. To understand the expression pattern of human *GREM1* and its possible contribution to neural differentiation, normalized counts and tSNE coordinates were employed for visualization using plots generated with ggplot2 package in R environment (Wickham, 2009). To investigate cell types expressing BMP antagonists, Pearson's chi-squared test of independence was performed in the R environment with chisq.test() function. Testing variables were gene expression categorized into high and low by median expression, and using cell type identifiers from the original scRNA-seq study (Fan et al., 2018). Scaled standardized residuals for each gene were used to plot the heatmap using the ComplexHeatmap package (Gu et al., 2016).

To examine the neural cell populations of the cortex expressing *Grem1* throughout developmental stages with single cell resolution in mice, we re-analyzed an scRNA-seq dataset with R version 4.0.3 (Yuzwa et al., 2017). Data were obtained from the Gene Expression Omnibus (GSE107122). We combined E11.5, E13.5, E15.5 and E17.5 together and analyzed with Seurat version 3.0 (Stuart et al., 2019). Data were firstly clustered by setting the dimension 1 to 16 and the resolution at 2 to obtain larger cell numbers within each cluster. Markers for each cluster were calculated through the FindAllMarkers() function, using receiver operating characteristic (ROC) analysis with minimum fraction set at 0.25 and log fold change set at 0.25. The top markers for each clusters were compared with the markers list from the original publication and clusters condensed to eight clusters according to the authors’ original markers: apical precursor – Sox2, Pax6, Hes1, Hes5, Pcna, Ung; proliferative RGC – Sox2, Pax6, Hes1, Hes5, Slc1a3, Ki67; nonproliferative RGC – Sox2, Pax6, Hes1, Hes5, Slc1a3; excitatory neuron – Tbr1, Tuj1, Satb2, Bhlhe22; newborn neuron – Tbr2, Tuj1, Tbr1, Foxp2, Rein; intermediate progenitor (IP)/excitatory neuron – Tbr2, Sstr2, Mfap4, Unc5d, Sema3c, Tuj1, Tbr1; apical IP – Tbr2, Gadd45g, Ngn1 (Neurog1), Ngn2 (Neurog2), Btg2, Sstr2, Mfap4; basal IP – Tbr2, Neurod1, Pam, Slc17a6, Sstr2, Mfap4 (Yuzwa et al., 2017). To examine the association between expression of genes of interest (BMP pathway and additional marker genes) and cell clusters, we categorized the expression of genes of interest into high and low expression groups at median expression. We then performed chi-squared tests using base function chisq.test() to look for correlations between gene expression and cell cluster. The standardized residuals for each gene were then visualized using a heatmap with ComplexHeatmap package (Gu et al., 2016).

### *In situ* hybridization

ISH analyses were performed on frozen mouse tissue samples using RNAscope technology (RNAscope 2.5 HD Detection Kit, Advanced Cell Diagnostics) following the manufacturer's instructions. Briefly, tissue sections were fixed by 4% paraformaldehyde (PFA) for 15 min, followed by incubation with an H_2_O_2_ solution (Pretreat 1 buffer) for 10 min at room temperature. Slides were boiled in a target retrieval solution (Pretreat 2 buffer) for 5 min, followed by incubation with a protease solution (Pretreat 3 buffer) for 30 min at 40°C. Slides were incubated with a mouse *Grem1* probe and a negative control probe (NM_011824.4, region 398-1359, catalogue number 314741 and 310043) for 2 h at 40°C, followed by successive incubations with signal amplification reagents. ISH staining was visualized with alkaline phosphatase substrate and Fast Red. Combined ISH/IHC was undertaken by first performing ISH, followed by IHC. For IHC following ISH, the sections were blocked with blocking buffer (X0909, Dako) and then incubated with a primary antibody (rabbit polyclonal anti-RFP, Rockland, 600-401-379, 1:1000) overnight at 4°C. The sections were washed in 1× phosphate-buffered saline 0.1% v/v Tween (PBST) three times and then incubated with HRP-conjugated secondary antibody (Vector Laboratories, PI-1000-1, 1:200) for 60 min at room temperature. IHC staining was visualized with Universal Elite ABC Kit (PK6101, Vector Laboratories) and DAB (K3468, Dako) and slides were counterstained with hematoxylin, dehydrated in ethanol, cleared in xylene and coverslipped. Cells expressing more than one ISH signal were regarded as positive for *Grem1* RNA.

### Real time RT-PCR

RNeasy mini kits (Qiagen) were used to isolate RNA from snap-frozen mouse brain tissues. RNA was reverse-transcribed into cDNA with cDNA master (Sigma-Aldrich). PCR amplification was performed with Kappa Sybr qPCR mix or Kappa Probe qPCR mix using QuantStudio7 (Applied Biosystems). The mouse *Gapdh* gene was used as an endogenous control. The following primers were used: TaqMan probes and primers – *gremlin 1* (IDT, Mm.PT.58.11631114), *Id1* (IDT,Mm.PT.58.6622645.g), *Id2* (IDT, Mm.PT.58.13116812.g), *Id3* (IDT, Mm.PT.58.29482466.g), *Id4* (IDT, Mm.PT.58.6851535); Sybr primers – *Gapdh*-forward AAGGTCATCCCAGAGCTGAA, Gapdh-reverse CTGCTTCACCACCTTCTTGA, *Ryr3*-forward TGCTGTCGCTTCCTTTGCTA, *Ryr3*-reverse CATCGATGGGGACGCTAGAC, *Lrrtm3*-forward TAGCAAATCAGGCTCCAGGG, *Lrrtm3*-reverse GAGTTCATGATGGACCCCACA, *MAG*-forward CCAGTACACCTTCTCGGAGC, *MAG*-reverse TCCGGCACCATACAACTGAC, *Gfap*-forward ACCGCATCACCATTCCTGTA, *Gfap*-reverse TGTGACTTTTTGGCCTTCCC, *Slc17a7*-forward CTTTTTGCGCAGTCGTCACA, *Slc17a7*-reverse GAGTATCCGACCACCAGCAG.

### Immunohistochemistry

To collect 14.5 dpc, 17.5 dpc and 20.5 dpc embryonic brains, euthanized pregnant dams immediately underwent cardiac perfusion with 4% PFA. Whole embryos at 14.5 dpc or dissected embryonic brains for 17.5 and 20.5 dpc were fixed in 4% PFA at 4°C overnight. Embryos and brains were then cryoprotected in 30% sucrose and frozen in OCT embedding medium. Then, 16 µm sections were cut using a Leica CM1900 cryostat. To make paraffin sections, samples were post fixed in 10% neutral buffered formalin overnight and processed. Then, 5 µm sections were cut using a Leica HM325 microtome. Sections were blocked with Protein Block Serum-Free (Dako) for 1 h at room temperature, incubated overnight with first antibody at 4°C, secondary antibody for 1 h at room temperature, and coverslipped with Vectashield Antifade Mounting Medium (Vector Laboratories). The following antibodies were used: anti-TBR1(Abcam, ab183032, 1:400), anti-NeuN (Abcam, ab104225, 1:500), anti-β tubulin III (Sigma-Aldrich, T5076, 1:400), anti-O4 (R&D Systems, MAB1326, 1:500), anti-GFAP (Dako, Z0334, 1:250), anti-FOXP2 (Abcam, ab16046, 1:10,000), anti-Ctip2 (Abcam, ab18465, 1:800), anti-CDP (Santa Cruz Biotechnology, sc-13024, 1:400), anti-BrdU (Abcam, ab6326, 1:600), anti-RFP (Rockland, 600-401-379, 1:1000), anti-Sox2 (Millipore, AB5603, 1:400), anti-Pax6 (Millipore, AB2237, 1:100), anti-TBR2 (Abcam, ab183991, 1:400), anti-Ki67 (Cell Signaling Technology, #12202, 1:200), anti-cleaved caspase 3 (Cell Signaling Technology, #9661, 1:400), anti-phospho SMAD1/5/8 (Millipore, AB3848-I, 1:200). Images were acquired on a Leica SP5 spectral scanning confocal microscope.

### Neural stem cell and progenitor cell culture

Before seeding cells, tissue culture dishes were coated with poly-D-lysine (PDL) (100 µg/ml) and laminin (10 µg/ml). Embryonic NSPCs were isolated from pregnant mice at 14.5 dpc. Dorsal telencephalons were dissected from each embryo in PBS, meningeal membranes removed and the tissue triturated to a single cell suspension. Cells were cultured in NeuroCult Proliferation Medium containing 20 ng/ml epidermal growth factor (EGF) (Stemcell Technologies). For differentiation assays, cells were seeded onto four-well chamber slides (Thermo Fisher Scientific, #NUN177399) in Neurocult Differentiation medium (Stemcell Technologies). To induce recombination or overexpression of *Grem1*, cells collected from Grem1^flox/flox^ embryos were infected with plenti-EF1-*Cre-2a-sfGFP-2a-puro*, plenti-EF1-*Grem1*-*2a-sfGFP-2a-puro* or plenti-EF1-*2a-sfGFP-2a-puro* lentivirus, and transduced cells were selected using puromycin for 5 days and used for experiments before passage 5. Lentivirus plasmids psPAX2 and MD2.G were transfected to 293T cells to generate lentivirus, and viral supernatant was concentrated using Amicon-Ultra 100k spin columns. To assess neurosphere forming ability, cells were seeded in 6-well uncoated plates (1.6×10^5^ cells per well) and cultured for 5 days. The number of neurospheres sized 50-100 µm and >100 µm was counted. For cell viability assays, NSPCs were seeded in 96-well coated plates (1×10^4^ cells per well) and RealTime-Glo™ MT Cell Viability Assay (Promega) was used with the continuous-read protocol at 0, 24, 52 and 72 h. Recombinant human BMP2 (Prospec) was added to the differentiation medium.

### Luciferase assay

BMP pathway activity was measured using the BMP response element luciferase reporter pGL3 BRE luciferase (Addgene plasmid #45126), internal control pRL/TK-luciferase reporter and the dual luciferase reporter assay kit (Promega). NSPCs were seeded in coated 24-well plates (1.6×10^5^ cells per well) and transfected with the reporter plasmids using xTremeGENE HP DNA transfection reagent (Roche). Cells were collected 48 h after transfection using Passive lysis buffer (Promega). The pGL3 empty vector was used as a control for BMP-independent changes in reporter activity.

### Western blot

Cell lysates were solubilized with M-PER Mammalian Protein Extraction Reagent (Thermo Scientific) containing complete protease and phosphatase inhibitors. Lysates were separated by SDS-PAGE and transferred to PVDF membranes (Millipore). After blocking with 5% nonfat skim milk in PBST for 30 min at room temperature, the membranes were incubated overnight at 4°C with anti-gremlin 1 antibody (R&D Systems, AF956, 1:1000) or anti-β-actin (Santa Cruz Biotechnology, sc-47778, 1:1000) in 0.5% nonfat skim milk in PBST. Membranes were washed with PBST and incubated with alkaline phosphatase-conjugated secondary antibodies (anti-rabbit IgG and anti-mouse IgG, GE Healthcare Life Sciences, NA934, NA931, 1:10,000) for 1 h at room temperature. Finally, the blot was visualized with Immobilon HRP substrate (Millipore) using a Chemi Doc XRS1 (Bio-Rad).

### Behavioral tests

Mice were submitted to Rotarod, open field, elevated plus maze and Y maze tests at the age of 7-10 weeks. Mice in their home cages were acclimatized to the behavior suite for at least 30 min before testing. The data was acquired blindly to the genotype.

Rotarod: animals were placed on the rotarod (Panlab/Harvard Apparatus) that linearly increased rotation speed from 4 to 40 rpm during a 120 s period. An accelerating protocol was employed to eliminate the need for habituation to the rotarod. This procedure was repeated for a total of three trials per mouse, separated by 15 min inter-trial intervals and the mean latency to fall from the rotarod in seconds was compared between wild-type littermate controls and *Grem1^cKO^* mice to assess motor coordination.

Open field test: the open field test was conducted in four identical square arenas (50×50×50 cm) surrounded by walls. Mice were individually placed in a corner of a clean arena and allowed to explore for 10 min. For the purpose of data collection, the arena was conceptually partitioned into two zones: a virtual center zone of 23×23 cm and a peripheral zone occupying the remaining area. A lower percentage of time spent in the center zone was used to indicate a higher level of anxiety.

Elevated plus maze test: the elevated plus maze consisted of a central square (8×8 cm) and four arms (29 cm long×8 cm wide, two open arms with no railing and two closed arms enclosed by a transverse wall 20 cm in height). A mouse was placed in the center of the central square facing the open arm and allowed to explore the maze apparatus for 10 min. The time spent in any of the open arms was recorded and used as a measure of anxiety.

Y-maze test: the apparatus consisted of three arms with an angle of 120° between each of the two arms. Each arm was 40 cm long×8 cm wide×15 cm high. Visual cues were placed on the walls of the mazes. The Y-maze test consisted of two trials separated by an inter-trial interval (ITI) to assess spatial recognition memory. The first trial (training) had a 5 min duration and allowed the mouse to explore only two arms of the maze, with the third arm (novel arm) being blocked. After 30 min ITI, the second trial was conducted, during which all three arms were accessible for 5 min. Trials were recorded using a ceiling-mounted camera and analyzed by a video analyzer (Ethovision XT, Noldus) to determine the period that each mouse spent in each arm of the maze.

### Statistics

All statistical analyses were performed using Graphpad prism 8, with methods and values summarized in each figure legend.

## Supplementary Material

Supplementary information
