# Supplementary material for: The BMP antagonist gremlin 1 contributes to the development of cortical excitatory neurons, motor balance and fear responses
Source: Development. 2021 Jul 12;148(14):dev195883. doi: 10.1242/dev.195883 (PMC8313862; doi:10.1242/dev.195883)
Supplement: Supplementary information [file develop-148-195883-s1.pdf]

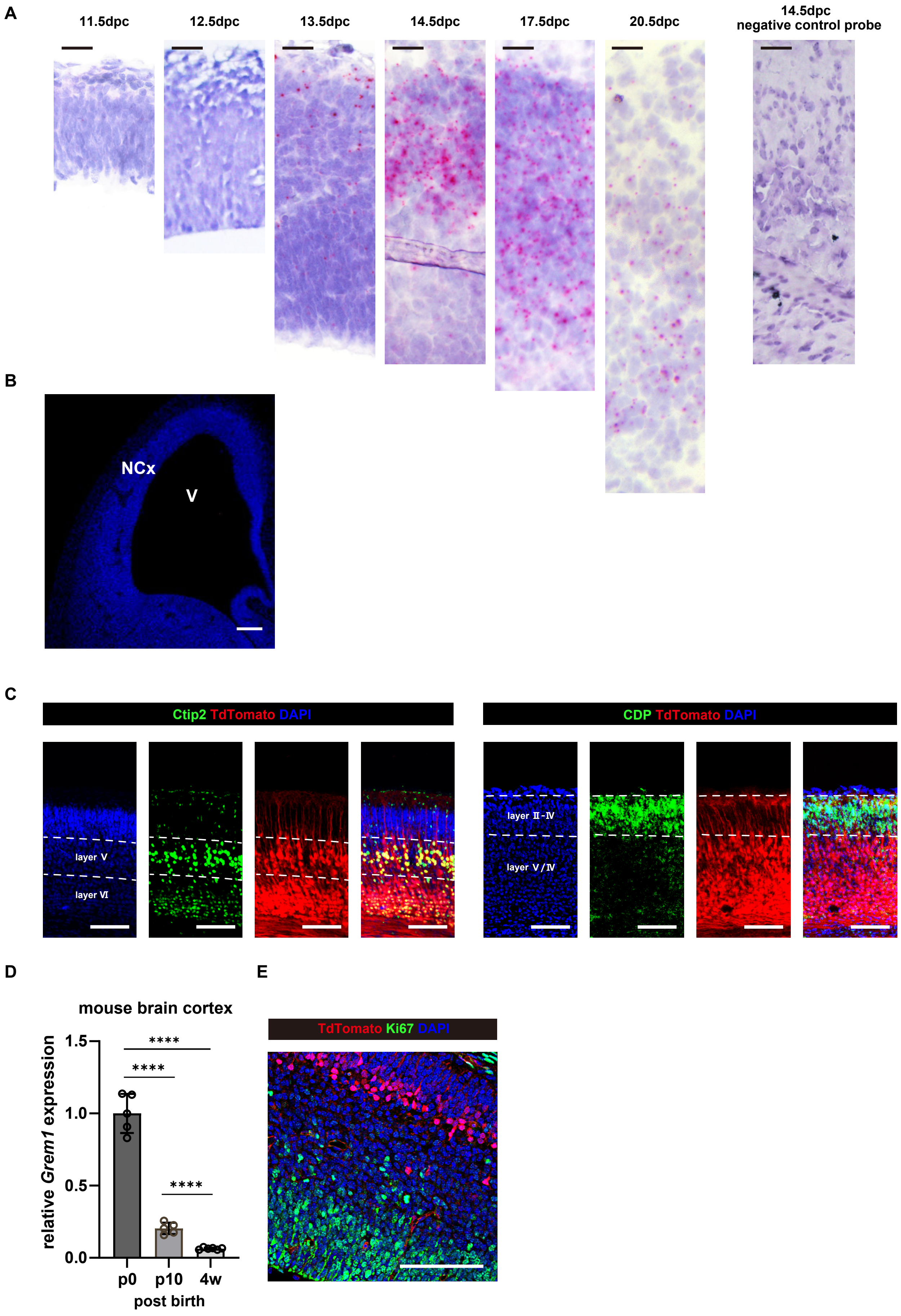
A
11.5dpc
12.5dpc
13.5dpc
14.5dpc
17.5dpc
20.5dpc
14.5dpc
negative control probe
B
NCx
V
C
Ctip2 TdTomato DAPI
layer V
layer VI
CDP TdTomato DAPI
layer II-IV
layer V/IV
D
mouse brain cortex
relative Grem1 expression
1.5
1.0
0.5
0.0
****
****
****
p0
p10
4w
post birth
E
TdTomato Ki67 DAPI

Fig. S1. *Grem1* starts to express at 13.5 dpc and is dramatically decreased after birth

(A) *Gremlin1* ISH of wild type mice cortex at stages spanning 11.5 to 20.5 dpc. No signal was detected with negative control probe at 14.5 dpc. Scale bar = 20 µm.

(B) Representative coronal images of immunofluorescence staining of 12.5 dpc telencephalon from *Grem1*-reporter (red) mice induced with tamoxifen at 11.5 dpc. Scale bar = 100 µm. LV, lateral ventricle ; NCx, neocortex

(C) Representative images of immunofluorescence staining of 20.5 dorsal telencephalon from *Grem1*-reporter (red) mice induced with tamoxifen at dpc13.5, DAPI (blue), and Ctip2 (green) for the left image, CDP (green) for the right image. Scale bar = 100 µm.

(D) *Grem1* mRNA levels normalized to *Gapdh* were determined using real time qRT-PCR in mouse cortical brain samples collected at p0, p10 and 4 weeks post-birth (n=4-6 mice/group, PCR conducted in triplicate) Columns, mean; bars, SD. One way ANOVA with Tukey's multiple test. ****p<0.0001

(E) Representative images of immunofluorescence staining of 14.5 dorsal telencephalon from *Grem1*-reporter (red) mice induced with tamoxifen at dpc13.5, Ki67 (green), DAPI (blue). Scale bar = 100 µm.

Development • Supplementary information

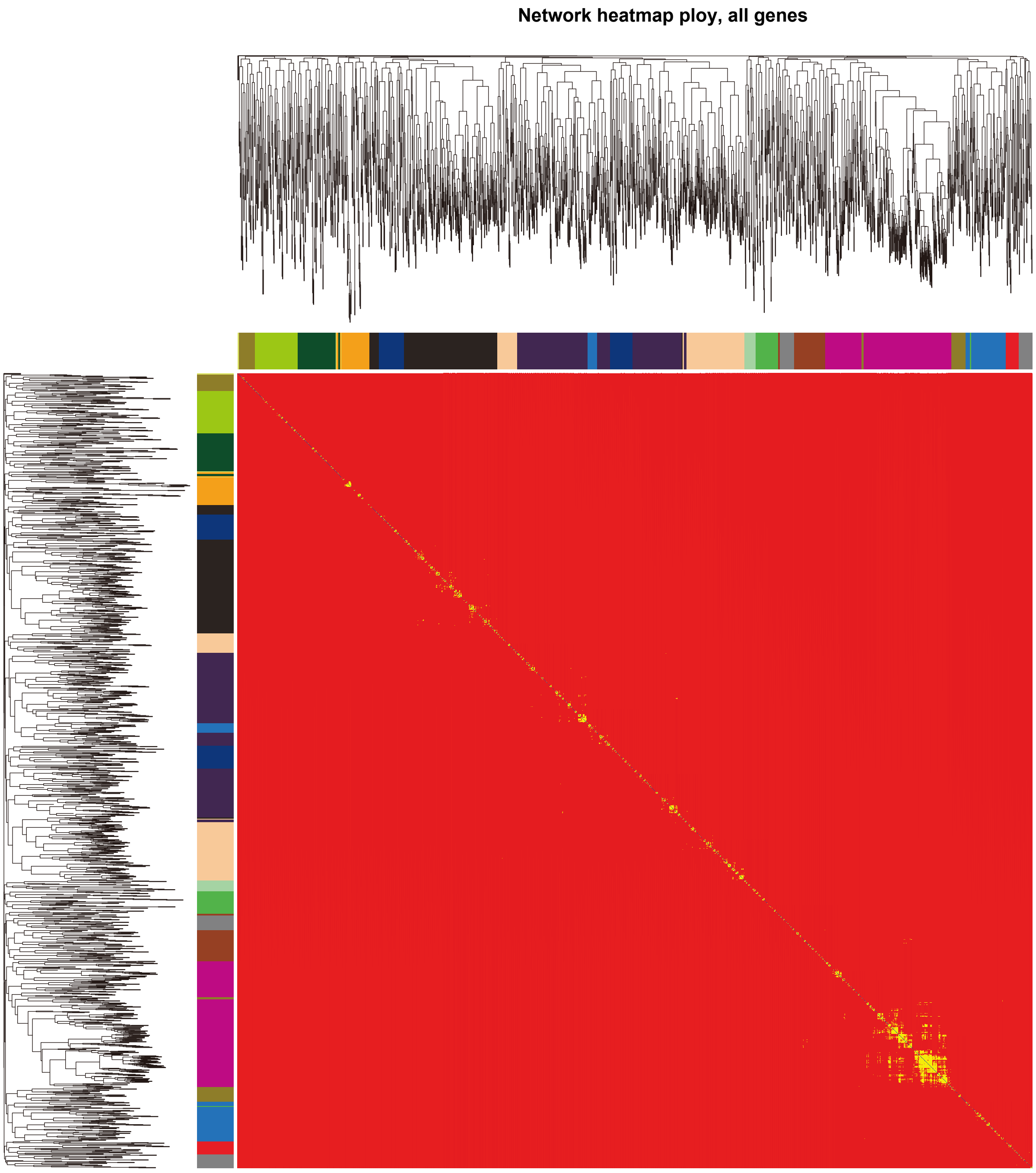

Fig. S2. Clustering of differentially expressed genes

A heat map was generated to visualise the unsupervised hierarchical clustering of correlation scores for gene expression of DEG in TdTomato+ cells. Yellow colour indicates closely related and red not related. The color bar between the hierarchical clustering tree and plotting fields denotes the module membership of each gene.

Development • Supplementary information

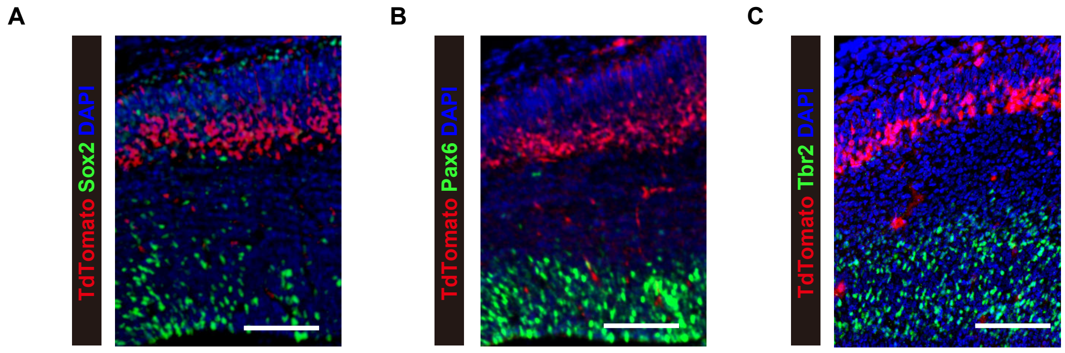

Fig. S3. Grem1-expressing cells are radial glial stem cell markers, Sox2 and Pax6 negative and an intermediate progenitor marker, Tbr2 negative

Representative images of immunofluorescence staining of 14.5 dpc neocortex from Grem1-reporter (red) mice induced with tamoxifen at dpc13.5, (A) Sox2 (green), DAPI (blue). (B) Pax6 (green), DAPI (blue). (C) Tbr2 (green), DAPI (blue). Scale bar = 100 μm.

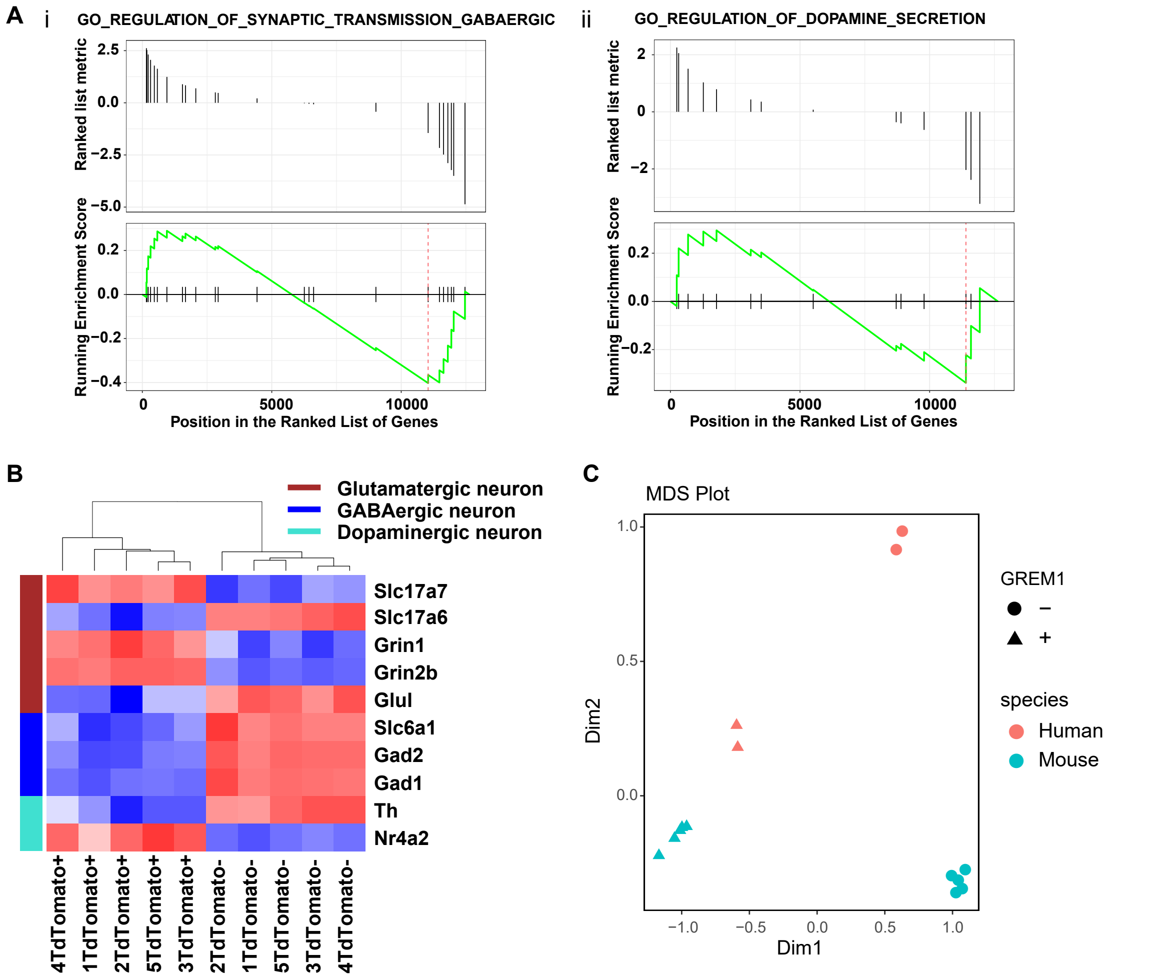

Fig. S4. Extended transcriptome analysis of bulk mRNAseq data from Grem1-expressing TdTomato+ and TdTomato- telencephalon cells at 14.5dpc

(A) Gene set enrichment analysis (GSEA) for genes involved in the regulation of **(i)** GABAergic synaptic transmissions and **(ii)** dopamine secretion that were differentially regulated between Grem1-expressing TdTomato+ and TdTomato- cells. Normalised enrichment score (NES) = -0.93, p = 0.75 and NES = -0.69, p = 0.87.

(B) Unsupervised clustering of Grem1-expressing TdTomato+ and TdTomato samples based on expression of representative markers for glutamatergic, GABAergic, and Dopaminergic neurons, in bulk mRNAseq data. Marker transcript expression is displayed as a heat map with high expression in red to low expression in blue.

(C) Multidimensional scaling plot comparison of transcriptome of TdTomato+ and TdTomato- cells isolated from 14.5dpc Grem1-reporter mice induced with tamoxifen at dpc13.5 and GREM1+ and GREM1- cells from scRNAseq of human mid-gestational cortex.

Development • Supplementary information

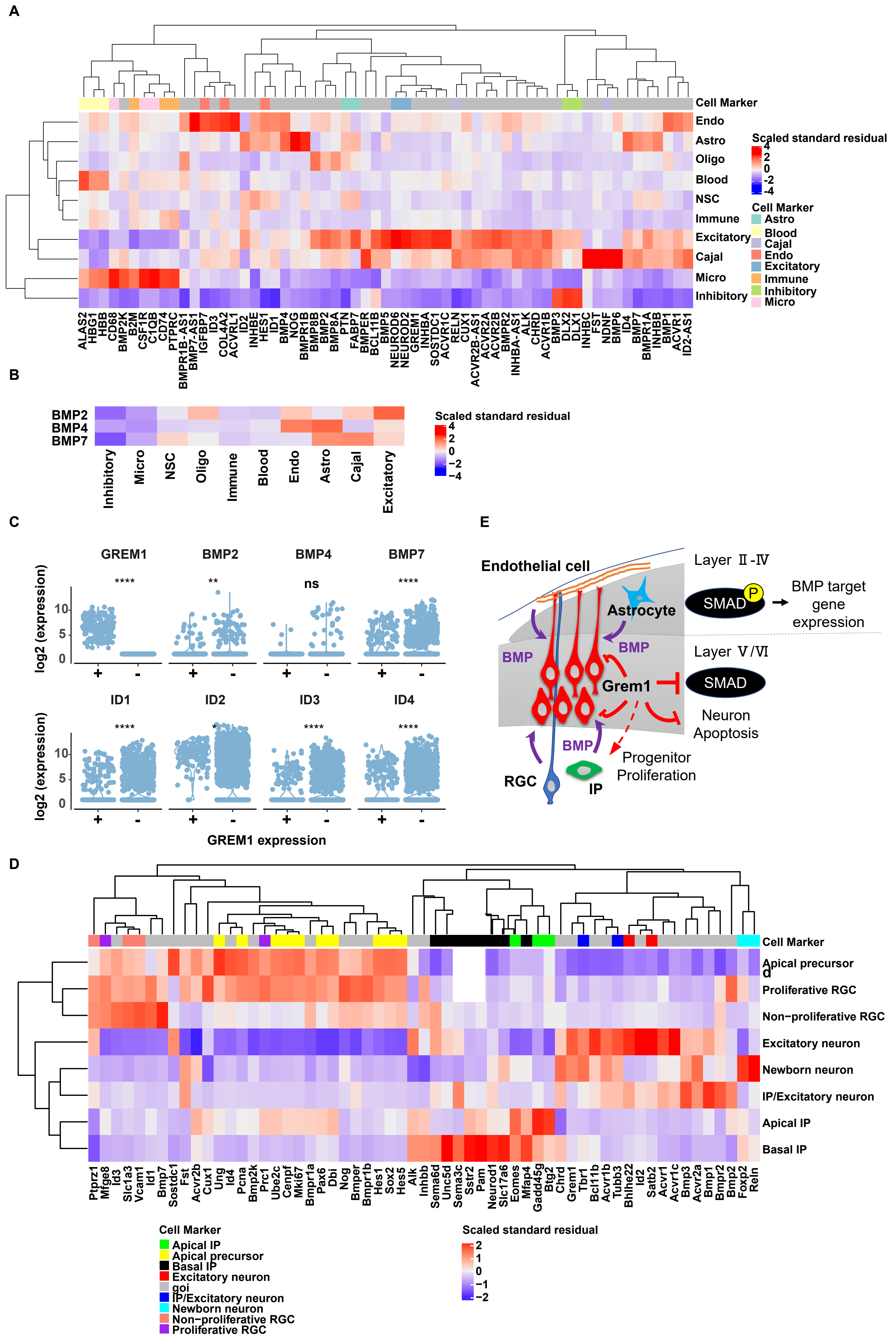

Development • Supplementary information

Fig. S5. Grem1 suppresses intrinsic BMP signaling in developing human excitatory neurons, likely via antagonism of paracrine BMP ligands.
(A) *GREM1* expression is associated with the excitatory neuronal lineage in the developing human cortex. Unsupervised clustering of human developmental brain cell populations from scRNAseq GSE103723 based on expression of lineage markers and transcripts encoding BMP ligands, BMP receptors (*BMPRI1/1b/ACVR2b,ALK, ACVR1b/1/1c/2a/BMPR2*), BMP antagonists (*FST, NOG, CHRD, BMPER, GREM1, SOSTDC1*), and BMP target genes (*ID1-4*). Cell type is indicated by colour legend as per tSNE plot shown in Figure 4E. Chi-square testing was used to evaluate the level of each transcript in each cell population and the resulting standardized residual values are depicted in the heat map.
(B) Heat map depicting expression of BMP ligands in human developmental brain cell populations from scRNAseq GSE103723. Cell types as per tSNE plot shown in Figure 4E. Chi-square testing was used to evaluate the level of each transcript in each cell population and the resulting standardized residual values are depicted in the heat map.
(C) *GREM1* expression inversely correlates with enrichment of *BMP2,7* and BMP target genes ID1, 3, 4 expression in human excitatory neurons in mid-gestation. Expression of transcripts encoding BMP ligands antagonised by *GREM1* and ID target genes in *GREM1*$^{+}$ and *GREM1*$^{-}$ cells is displayed from human scRNAseq GSE103723. One way ANOVA with Tukey's multiple test. ns p>0.05, *p<0.05, **p<0.01,****p<0.0001
(D) *Grem1* expression is associated with the excitatory neuronal lineage in the developing mouse cortex. Unsupervised clustering of mouse embryonic brain cell populations from scRNAseq GSE107122 based on expression of lineage markers and transcripts encoding BMP ligands, BMP receptors (*Bmpri1/1b/Acvr2b,Alk, Acvr1b/1/1c/2a/Bmpr2*), BMP antagonists (*Fst, Nog, Chrd, Bmper, Grem1, Sostdc1*), and BMP target genes (*Id1-4*). Cell type is indicated by colour legend. Chi-square testing was used to evaluate the level of each transcript in each cell population and the resulting standardized residual values are depicted in the heat map. RGC, radial glial cells ; IP, intermediate progenitors ; Str, striatum **(E)** Schematic depicting BMP signaling regulation by *GREM1* in the developing cortex. Our analyses of scRNA data suggest BMP ligands are expressed by different cell populations in the developing cortex to the excitatory neuron populations that express Grem1/GREM1. Expression of the BMP target genes, ID1,3,4 was inversely correlated with *GREM1*-expression in excitatory neurons suggesting *GREM1* acts locally to antagonize BMP signaling. Cortical deletion of *Grem1* resulted in increased apoptosis and phospho-SMAD1,5,8 in Ctip2+ layer V/VI neurons, but not upper cortical layers consistent with Grem1 acting locally in layer V/VI to antagonize BMP signaling and enhance neuron survival. It is possible that Grem1 also promotes proliferation of progenitors (dashed red line)

Development • Supplementary information

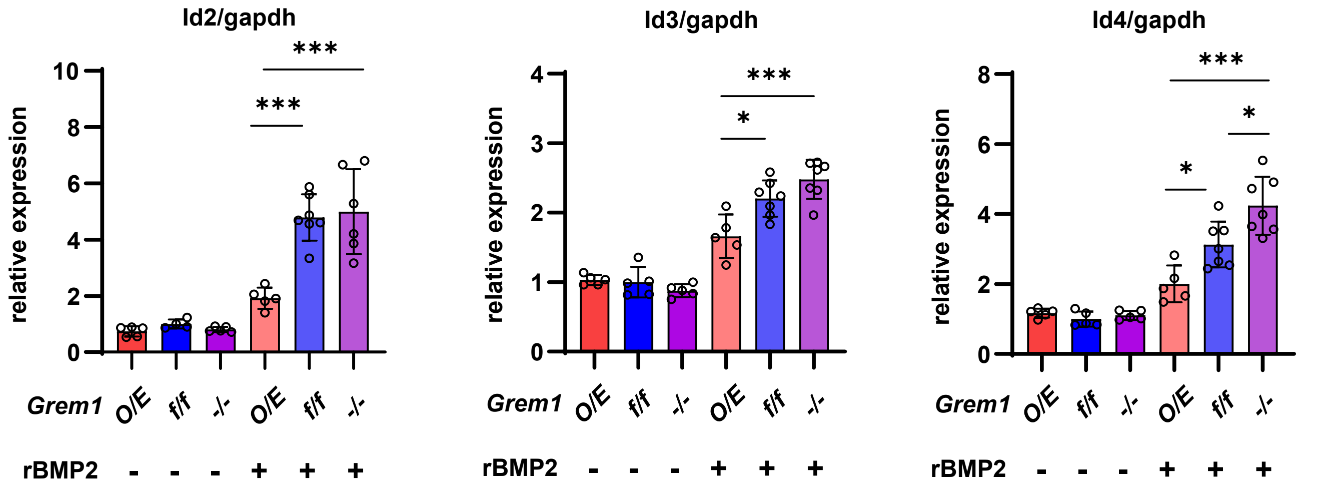

Fig. S6. Grem1 acts to antagonize BMP signaling in NSPCs resulting in altered downstream transcript levels

Transcript levels of BMP target genes, Id2, Id3 and Id4, normalized to Gapdh in Grem1flox/flox, Grem1-/- and Grem1O/E NSPC treated with vehicle or rBMP2 for 24h. Results from 5 independent experiments performed in triplicate. Columns, mean; bars, SD. One way ANOVA with Tukey's multiple test. *p<0.05, ***p<**0.001**

Development • Supplementary information

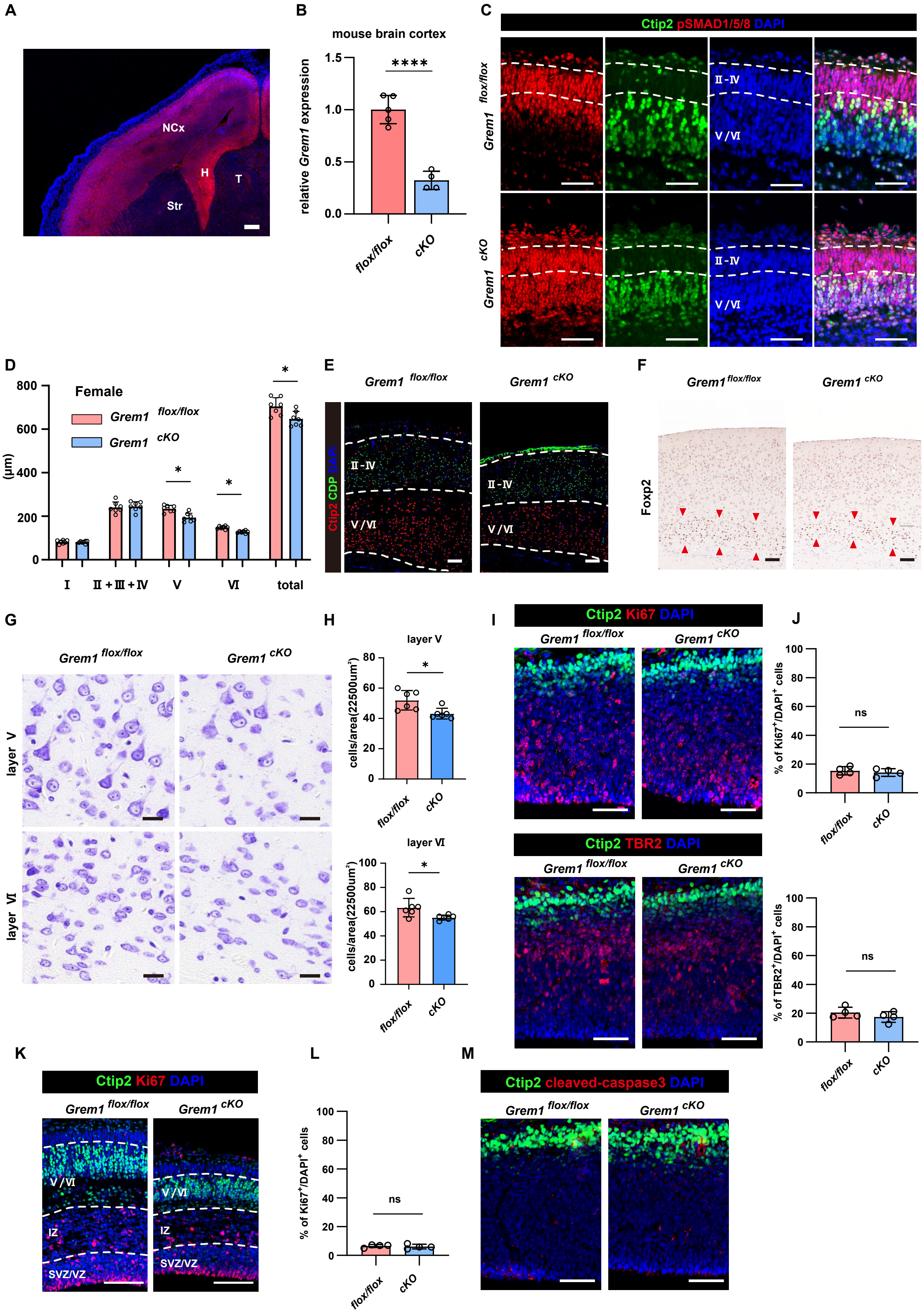

Development • Supplementary information

Fig. S7. Extended morphological, immunostaining and transcript analysis of *Grem1^{cKO}* mice and littermate controls

(A) Representative coronal section of TdTomato+ (red) cells in the dorsal telencephalon at 14.5dpc in *Emx1-cre ; Rosa26LSLTdtomato* mouse brain. This cre-driver is broadly active across the telencephalon region in which *Grem1* is highly expressed. Scale bar = 100 µm. NCx, neocortex ; H, hippocampus ; Str, striatum ; T, thalamus

(B) *Grem1* mRNA levels normalized to *Gapdh* were determined using real time qRT-PCR in mouse cortical brain samples collected at postnatal day 0 from *Grem1^{cKO}* mice and littermate controls n=4 biological replicates. Columns, mean; bars, SD. t-test. ****p<0.0001

(C) Representative images of immunofluorescence staining of cortex of Grem1cKO mice and littermate controls at 20.5 dpc with layer Ⅴand Ⅵ with marker Ctip2 (green),

phosphorylated smad1/5/8 (red), and DAPI (blue). n = 4 Scale bar = 100 µm.

(D) Quantification of cortical layer thickness compared between age and sex matched littermates at 10 weeks of age. Female, n=7 control and n=7 *Grem1^{cKO}*, t-test, *p<0.05. **(E)** Representative images of immunofluorescence staining of cortex of *Grem1^{cKO}* mice and

littermate controls at 10 weeks of age to visualise layer Ⅱ-Ⅳ with marker CDP (green),

layer Ⅴand Ⅵ with marker Ctip2 (red), and DAPI (blue). n = 3 Scale bar = 100 µm.

(F) Representative images of immunohistochemical staining of cortex of *Grem1^{cKO}* mice and

littermate controls at 10 weeks of age to visualise layer Ⅵ marker Foxp2 IHC (shown

in red arrowheads). n = 3 Scale bar = 100 µm.

(G) Representative histological images of neocortical layer V and Ⅵ from *Grem1^{cKO}* mice

and *Grem1$^{flox/flox}$* littermate controls at 10 weeks of age using Nissl. Scale bar = 20um.

(H) Quantification of (G) showing the cell number per area of layer V and Ⅵ using Nissl

in 2 HPF of 3 biological replicates. Columns, mean; bars, SD. t-test. *p<0.05. Scale bar = 20um. **(I)** Representative images of immunofluorescence staining of cortex of *Grem1^{cKO}* mice and in 2 HPF of 3 biological replicates. Columns, mean; bars, SD. t-test. *p<0.05. Scale bar = 20um.

(I) Representative images of immunofluorescence staining of cortex of Grem1cKO mice and littermate controls with layer Ⅴand Ⅵ marker Ctip2 (green), Ki67 (red), and DAPI (blue) / Ctip2 (green), Tbr2 (red), and DAPI (blue) at 14.5 dpc. n = 4 Scale bar = 50 µm.

(J) Quantification of (I) showing the percentage of Ki67+ or Tbr2+cells that were also DAPI+ in 4 biological replicates. t-test. ns : not significant.

(K) Representative images of immunofluorescence staining of cortex of Grem1cKO mice and littermate controls with layer Ⅴand Ⅵ marker Ctip2 (green), Ki67 (red), and DAPI (blue) at 20.5 dpc. n = 4 Scale bar = 100 µm.

(L) Quantification of (K) showing the percentage of Ki67+ that were also DAPI+ in 4 biological replicates. t-test. ns : not significant.

(M) Representative images of immunofluorescence staining of cortex of Grem1cKO mice and littermate controls with layer Ⅴand Ⅵ marker Ctip2 (green), cleaved caspase3 (red), and DAPI (blue) at 14.5 dpc. n = 4 Scale bar = 50 µm.

Development • Supplementary information

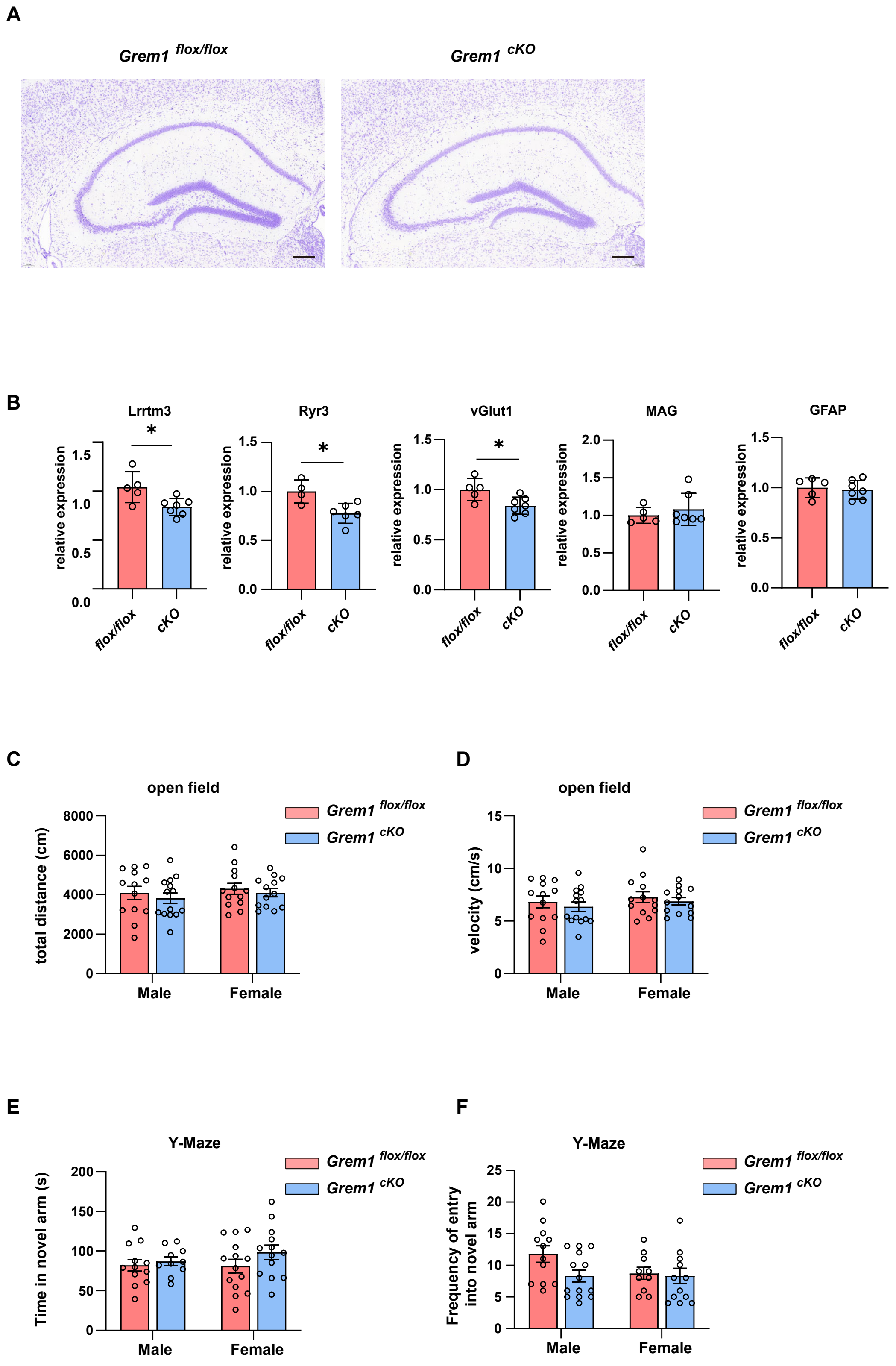

Development • Supplementary information

Fig.S8 Memory is not impaired in *Grem1*^{cKO} mice compared to *Grem1*$^{flox/flox}$ littermate ontrols

(A) Representative histological images of hippocampus from *Grem1*^{cKO} mice and *Grem1*$^{flox/flox}$ littermate controls at 10 weeks of age using Nissl. 8 pairs of males and 7 pairs of females were analyzed. Scale bar = 200 µm.

(B) Transcripts from the *Id1* associated gene cluster (Fig2E), *Ryr3* and *Lrrtm3* , and differentiation markers, *vGlut1* for excitatory neurons, *GFAP* for astrocytes, and *MAG* for oligodendrocytes were evaluated in *Grem1*^{cKO} and *Grem1*$^{flox/flox}$ littermate control cortex at p10 using real time qRT-PCR. *Gene* levels were normalized to *Gapdh*. n=4-6 biological replicates. Columns, mean; bars, SD. unpaired t-test, two tailed. *p<0.05

(C,D) Open field test. Behavior was compared between age and sex matched *Grem1*^{cKO} mice and *Grem1*$^{flox/flox}$ littermate controls at 7-10 weeks of age. Male, n=12 control and n=10 *Grem1*^{cKO}, Female, n=14 control and n=13 *Grem1*^{cKO}. unpaired t-test, two tailed. **(C)** The total distance moved. **(D)** The mean velocity. Columns, mean; bars, SEM.

(E,F) Y maze test. Behavior was compared between age and sex matched *Grem1*^{cKO} mice and *Grem1*$^{flox/flox}$ littermate controls at 7-10 weeks of age. Male, n=12 control and n=10 *Grem1*^{cKO}, Female, n=14 control and n=13 *Grem1*^{cKO}. unpaired t-test, two tailed. **(E)** Cumulative duration spent in new arm of Y maze. **(F)** The number of entries to new arm of the Y maze. Columns, mean; bars, SEM.

Development • Supplementary information
